# Supplementary material for: Large protein databases reveal structural complementarity and functional locality
Source: Nat Commun. 2025 Aug 25;16:7925. doi: 10.1038/s41467-025-63250-3 (PMC12378989; doi:10.1038/s41467-025-63250-3)
Supplement: Supplementary file 1 — Supplementary Information [file 41467_2025_63250_MOESM1_ESM.pdf]

Large protein databases reveal structural complementarity and functional locality

Supplementary Information

Paweł Szczerbiak, Lukasz Szydlowski, Witold Wydmański,
P. Douglas Renfrew, Julia Koehler Leman, Tomasz Kosciolek*

*Corresponding author: t.kosciolek@sanoscience.org

Structural clustering 1
- **First stage: MIP database** 2
  - **The largest cluster of MIP novel folds** 4
- **First stage: highquality_clust30 dataset** 6
- **Second stage: final dataset** 9

Structure space 14
- **PaCMAP grid search** 14
- **Visualizations** 18
  - **Plots for unnormalized Geometricus representations** 19
- **Other databases** 21

Functional annotations 27
- **deepFRI v1.1** 27
- **Validation using E. coli proteome** 27
- **Visualizations** 29
  - **Plots for unnormalized Geometricus representations** 30
- **Top COG categories** 32

Cluster heterogeneity 33
Taxonomy analysis 40
References 42

Structural clustering

Similarly to reference (1) we used Foldseek to remove structural redundancy and find cluster representatives. In all cases, we used cov-mode = 0 (coverage of query and target). By cluster we understand a group of similar structures of cardinality at least 2 (the rest are singletons). We utilized a two-stage procedure (see Fig. 1a in the main text):

- First stage: cluster each dataset independently i.e. AFDB50 (already done in reference (1)), highquality_clust30 (high quality predictions from ESMAtlas with pTM and pLDDT > 0.7 clustered at 30% sequence similarity level) [https://github.com/facebookresearch/esm/blob/main/scripts/atlas/README.md], and MIP (2) (mostly single-domain structures already filtered at 30% sequence identity

level). Foldseek parameters (e-value and coverage) have been tuned separately for each dataset (see subsequent paragraphs).

- Second stage: gather cluster representatives and MIP singletons (we exclude AFDB and ESMAtlas singletons from the reasons described in the main text – see Methods section), and cluster this set with Foldseek.

First stage: MIP database

To find optimal Foldseek parameters for the MIP database, which comprises short (between 40 and 200 residues) mostly single-domain proteins, we chose e-values = [0.1, 0.01, 0.001, 0.0001] and structural alignment overlap c = [0, 0.9]. The second value for the c parameter was chosen to align with the procedure in reference (1). For similar reasons, we did not consider more extreme e-values.

Non-zero coverage parameter c results in more clusters (and singletons) since we require that two given structures must be not only similar but also of comparable size – see Supplementary Figure 1. It is also clear from the plot that the number of singletons scales almost linearly with e-value. For the number of clusters it is more complicated but we can notice that e-value = 0.001 provides the biggest number of clusters for c = 0; for c = 0.9 we can observe a small decrease with e-value with a sharp drop at 0.0001. As a consequence, mean cluster size also decreases with e-value and c parameter – see Supplementary Figure 2. The same concerns the maximum cluster size for c = 0.9; interestingly, for c = 0 we can notice an increase in e-value with a large drop, again at 0.0001. Those effects are probably heavily dataset-dependent but, in general, e-values between 0.1 and 0.001 should provide a reasonable tradeoff between the number of clusters and their size. In Supplementary Figure 3 we show that a non-zero c parameter is critical if we need to ensure cluster consistency. For c = 0 representative structures might be short (two times smaller in this case) and not grasp the full diversity of the clusters they represent. Interestingly, for c = 0.9 the distribution of the longest structure size to representative structure size almost does not depend on e-value.

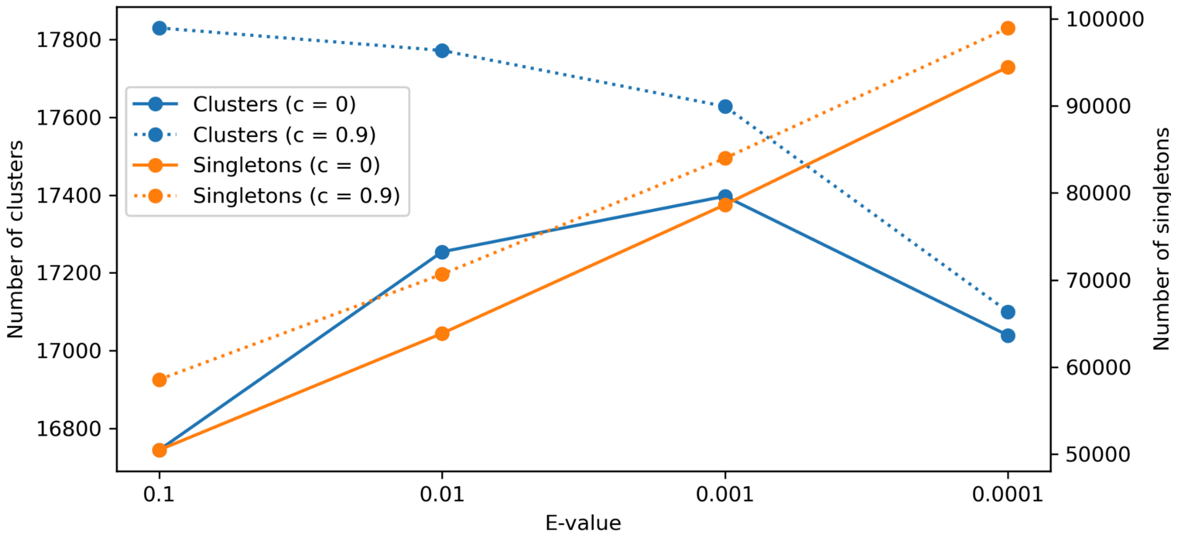

Supplementary Figure 1: Number of clusters (left y-axis) and singletons (right y-axis) as a function of e-value stratified by coverage parameter, c.

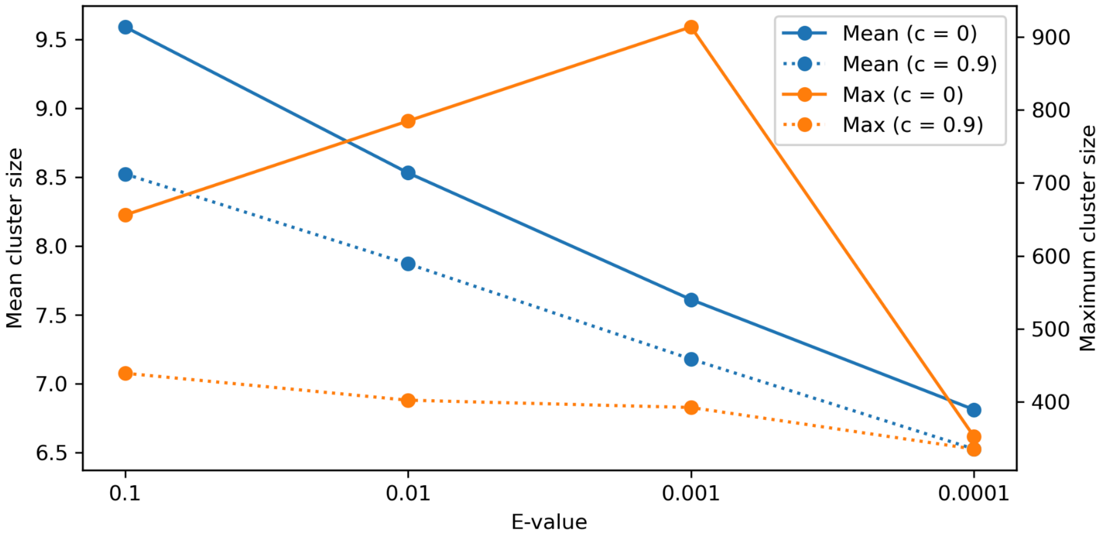

Supplementary Figure 2: Mean cluster size (left y-axis) and maximum cluster size (right y-axis) as a function of e-value stratified by coverage parameter, c. Note that the standard deviation is not meaningful here because the distribution of cluster sizes does not follow a normal distribution.

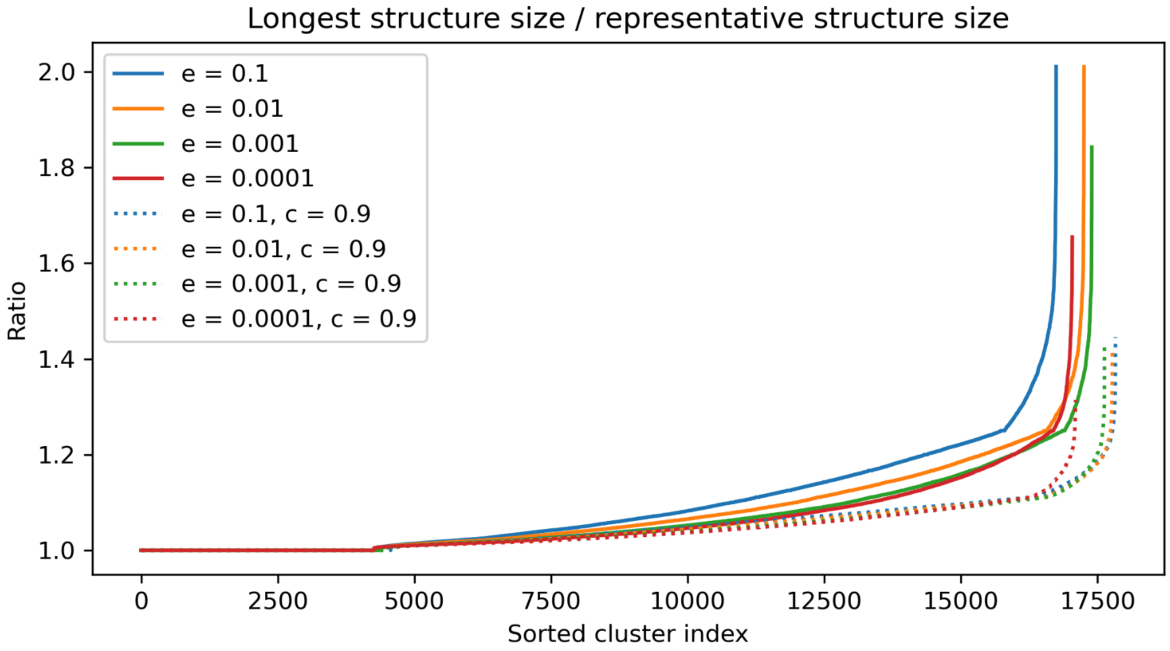

Supplementary Figure 3: Ratio (y-axis) between the longest and representative structure sizes for a given cluster (x-axis). Cluster indices in the x-axis are sorted based on the value on the y-axis. Each line corresponds to a different e-value, e, and coverage parameter, c.

To estimate structural similarity across clusters we superimposed all structures within each cluster with US-align – see Supplementary Figure 4 and Supplementary Figure 5. According to expectations, the TM-score increases with e-value, however, the differences are not large (at least for e-value ≥ 0.01). Interestingly, coverage, c, does not influence structural similarity so much in this case. Still, we can observe a larger discrepancy between minimum, mean, and maximum TM-score distribution for c = 0 as compared to c = 0.9.

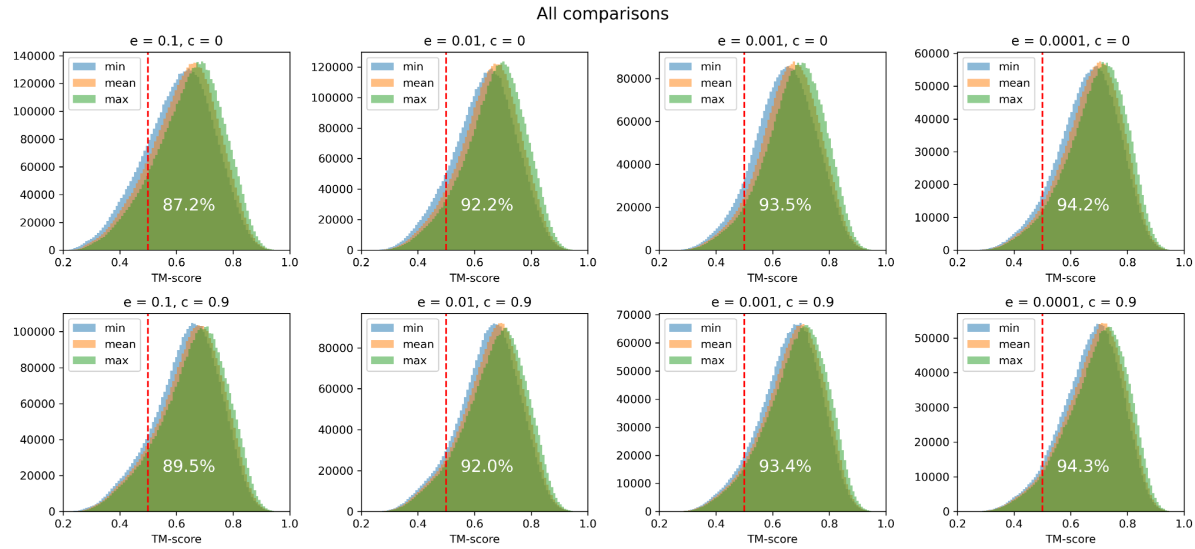

Supplementary Figure 4: Distribution of minimum, mean, and maximum TM-score between each two structures (excluding identities) within a given cluster for all clusters. Each panel corresponds to a different e-value, e, and coverage parameter, c, combination. Numbers in white denote percentages of points with mean TM-score ≥ 0.5 (red vertical line).

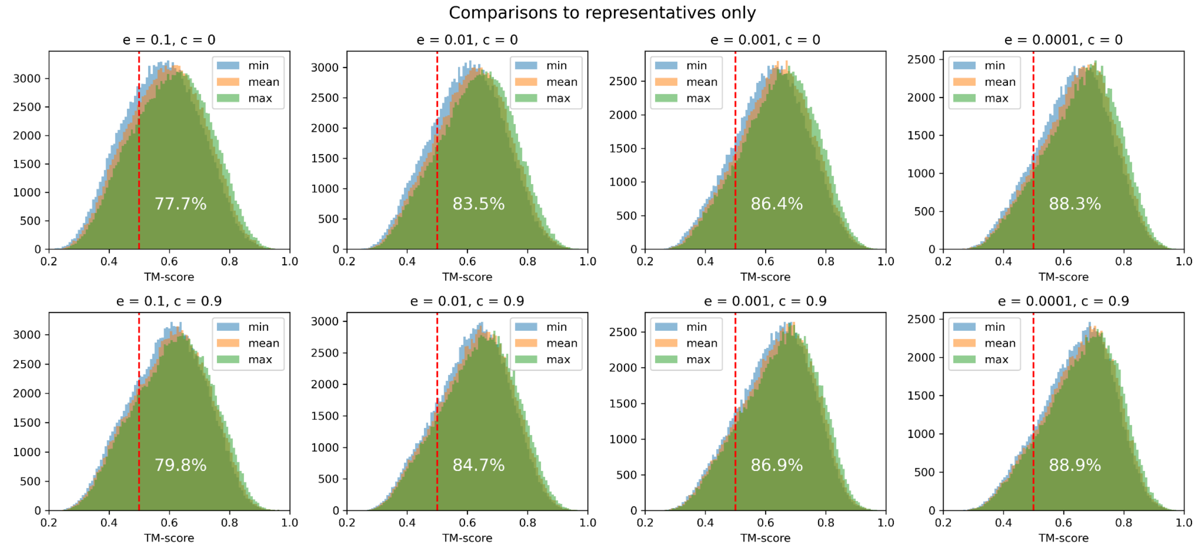

Supplementary Figure 5: Distribution of minimum, mean, and maximum TM-score between each structure and representative structure (excluding identities) within a given cluster for all clusters. Each panel corresponds to a different e-value, e, and coverage parameter, c, combination. Numbers in white denote percentages of points with mean TM-score ≥ 0.5 (red vertical line).

The largest cluster of MIP novel folds

As a final verification of foldseek clustering, we checked whether we could identify the largest cluster of MIP novel folds (Supplementary Figure 53 in reference (2)). Surprisingly, for each combination of foldseek parameters that we tested (most importantly, all e-values), the cluster comprised 105 structures including all 87 found in the aforementioned MIP cluster – see Supplementary Figure 6. What is even more interesting, the additional 18 structures demonstrate very high TM-score within themselves and against the 87 ones (Supplementary Figure 7). **It proves that Foldseek is indeed a reliable protein structure**

clustering method. The reason why those additional structures had not been taken into account when constructing the MIP novel folds is the prefiltering step. Namely, we required the DMFold and Rosetta models to both have max TM-score against PDB90 ≥ 0.5 which was not fulfilled (sometimes at a marginal level e.g. 0.01). This indicates that using rigid thresholds on similarity metrics for identifying potential novel folds may not be the most advantageous approach.

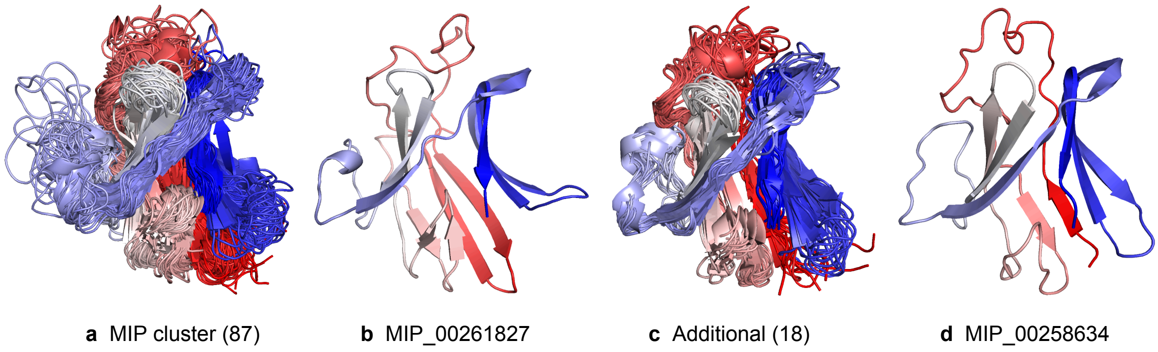

Supplementary Figure 6: Foldseek cluster that overlaps with the largest cluster of MIP novel folds. (**a**): Structures that have been found also in the MIP cluster. (**b**): Example structure in **a**. (**c**): Additional structures found in the Foldseek cluster. (**d**): Example structure in **c**.

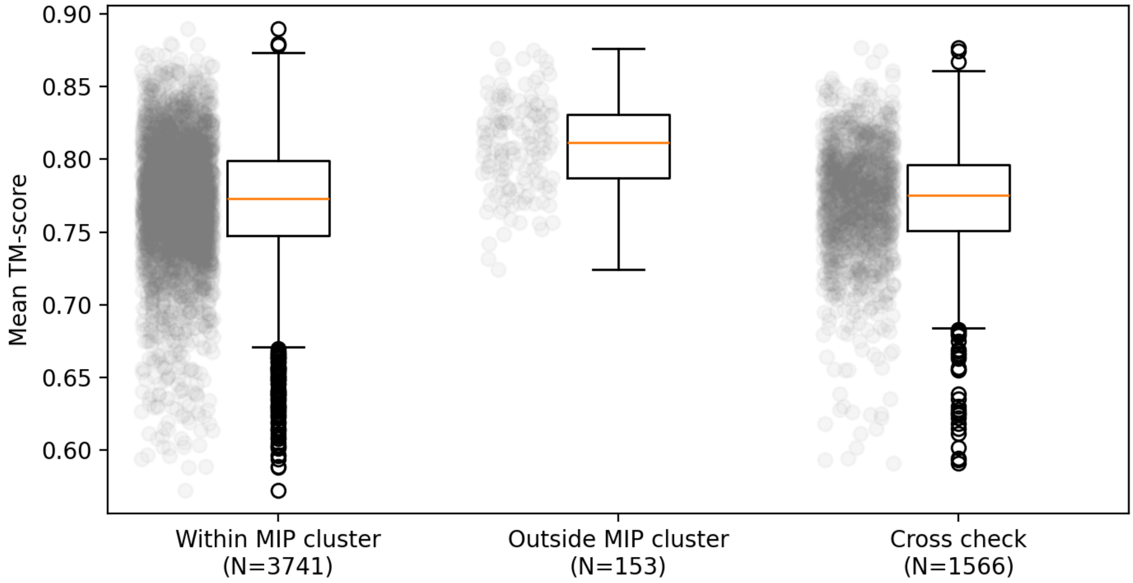

Supplementary Figure 7: Mean TM-score between all superimposed structures within a given group i.e. (from left to right) superposition of structures present in the largest cluster of MIP novel folds (Supplementary Figure 6a), superposition of additional structures not present in the previous group (Supplementary Figure 6c), crossed superposition between first and second group. Orange lines represent median values, whereas black lines show interquartile ranges (IQR); whiskers indicate variability outside the upper and lower quartiles, and circles represent outliers. Number of data points for each box is shown below the x-axis. Individual data points for each box are shown to the left.

In summary, the optimal set of analyzed hyperparameters that provides high consistency and similarity within clusters and a satisfactory number of clusters/singletons for the MIP database is **e-value = 0.001** and **c = 0.9**. The key observation is, however, that many features (including identification of compact clusters) do not depend so strongly on e-value.

First stage: highquality_clust30 dataset

Similarly, we utilized the same procedure to cluster a high-quality subset of ESMAtlas, i.e. highquality_clust30. Results are presented in plots Supplementary Figure 8–Supplementary Figure 10. We might notice many similarities to the MIP database clustering. Interestingly, the mean cluster size (which can be treated as a measure of structural redundancy) is a few times larger as compared to the MIP.

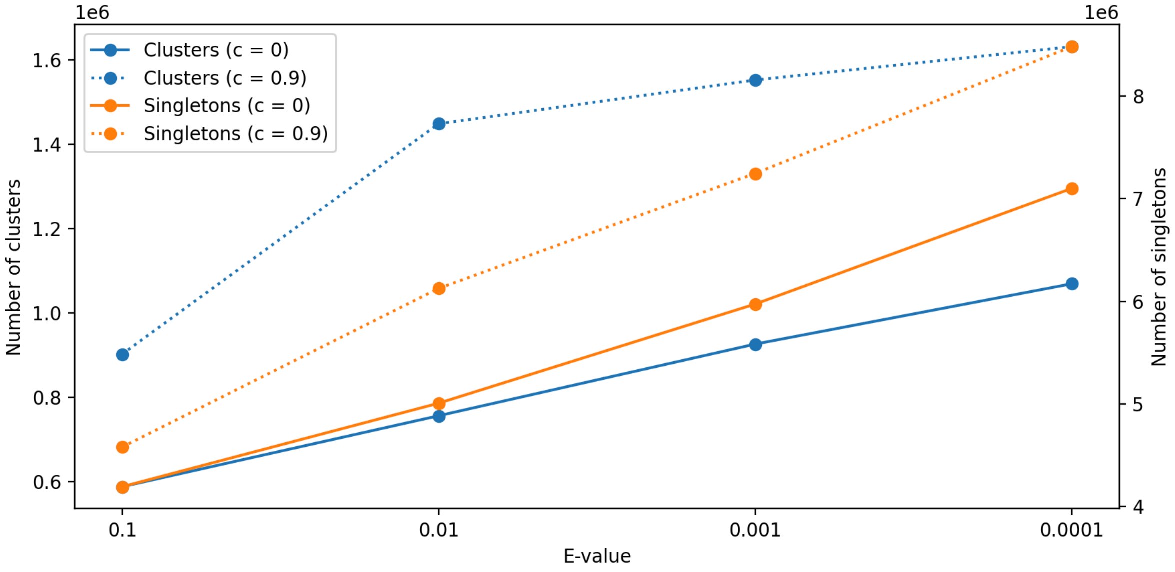

Supplementary Figure 8: Number of clusters (left y-axis) and singletons (right y-axis) as a function of e-value stratified by coverage parameter, c.

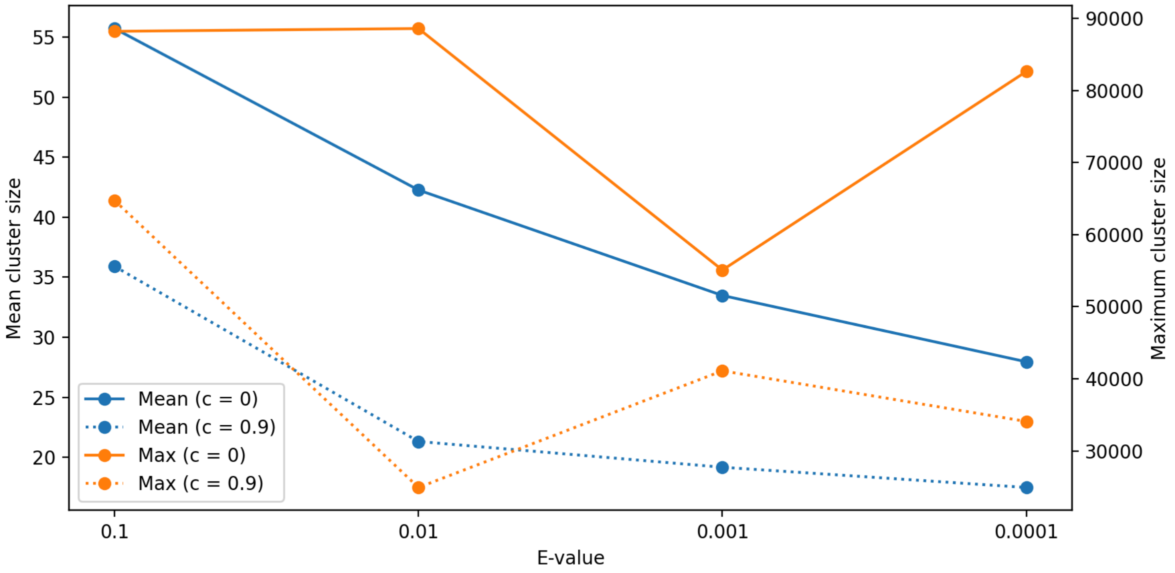

Supplementary Figure 9: Mean cluster size (left y-axis) and maximum cluster size (right y-axis) as a function of e-value stratified by coverage parameter, c. Note that the standard deviation is not meaningful here because the distribution of cluster sizes does not follow a normal distribution.

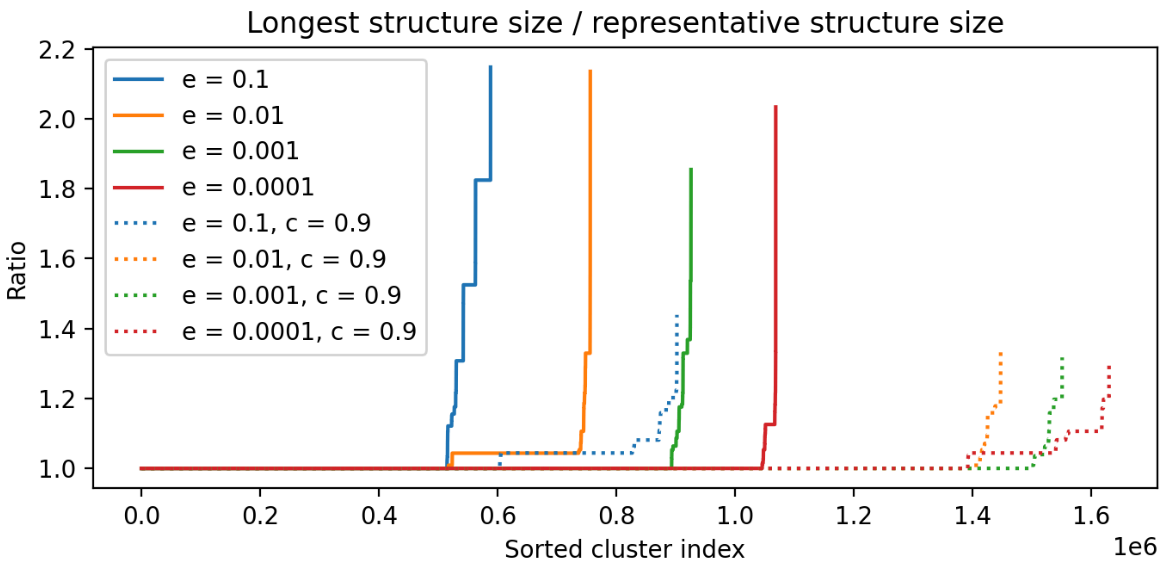

Supplementary Figure 10: Ratio (y-axis) between the longest and representative structure sizes for a given cluster (x-axis). Cluster indices in the x-axis are sorted based on the value on the y-axis. Each line corresponds to a different e-value, e, and coverage parameter, c.

For further investigation, we chose three combinations i.e. e-value = 0.01, 0.001, 0.0001, and c = 0.9. When it comes to the overall TM-score distribution (see Supplementary Figure 11), all e-values provide satisfactory outcomes. Surprisingly, the TM-score between the representative and all the other structures for the top 12 largest clusters favors e-value = 0.01 (see Supplementary Figure 12). In general, however, the number of poor (median TM-score between representative structure and all the other structures per cluster between 0.4 and 0.5) and very poor clusters (median TM-score smaller than 0.4) decreases with increasing e-value – see Supplementary Figure 13 and Supplementary Table 1.

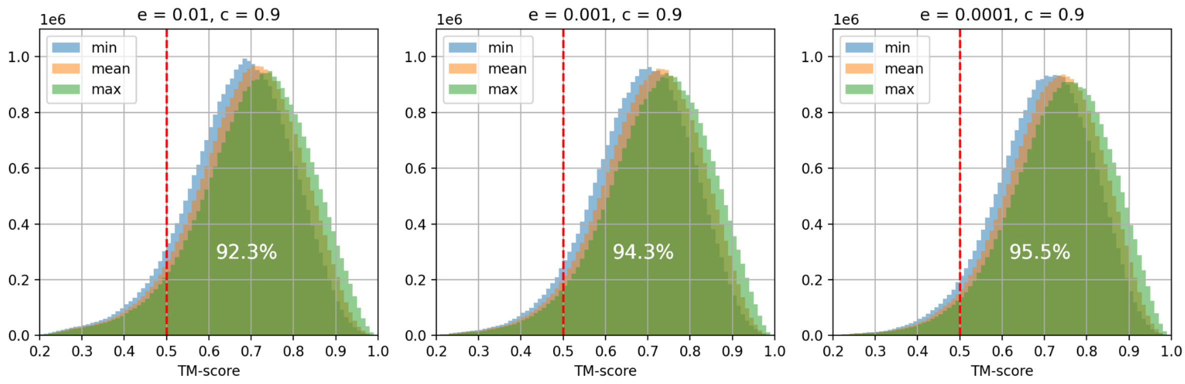

Supplementary Figure 11: Distribution of minimum, mean, and maximum TM-score between each structure and representative structure (excluding identities) within a given cluster for all clusters. Each panel corresponds to a different e-value, e, and coverage parameter, c, combination. Numbers in white denote percentages of points with mean TM-score ≥ 0.5 (red vertical line).

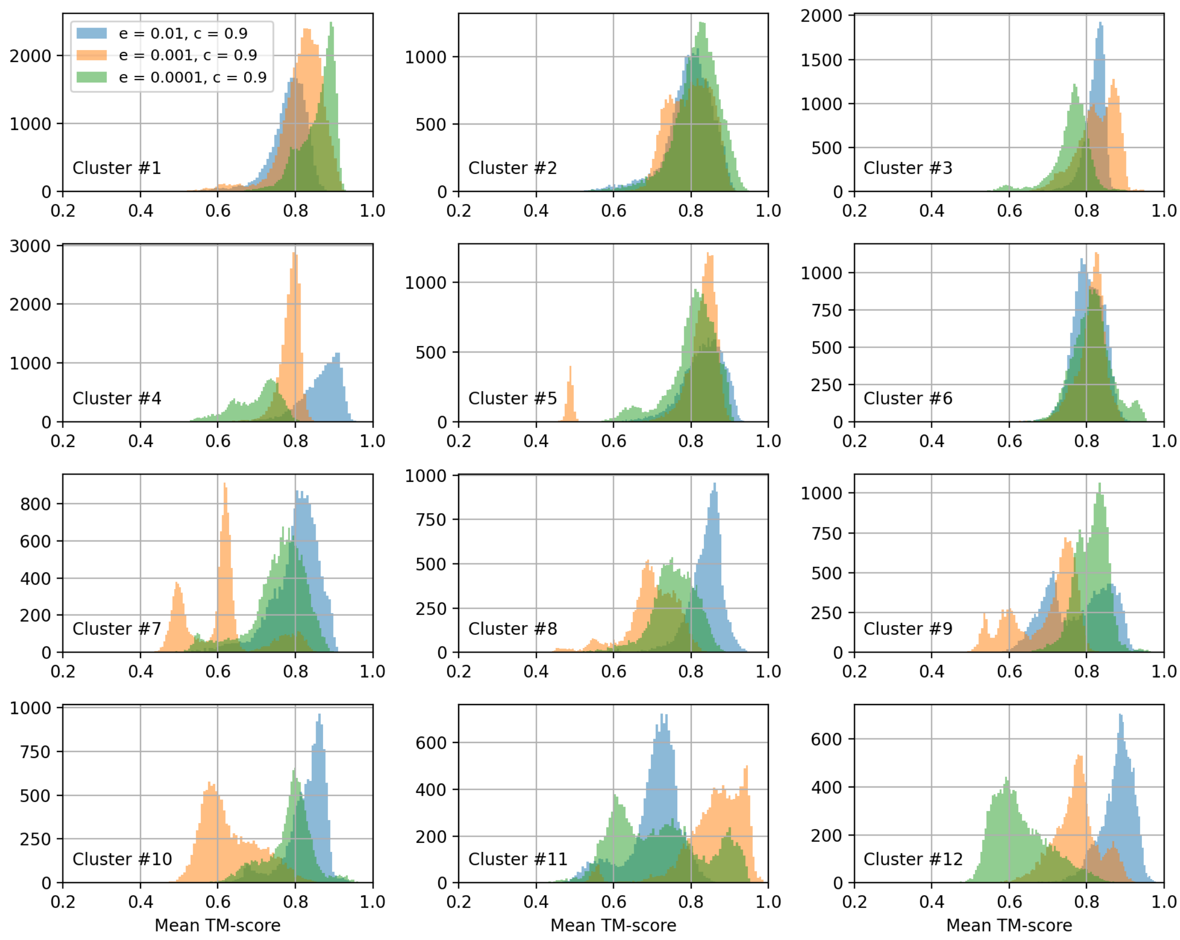

Supplementary Figure 12: Distribution of mean TM-score between each structure and representative structure (excluding identities) within a given cluster for the top 12 largest clusters. Each color corresponds to a different e-value, e, and coverage parameter, c, combination (see top left legend).

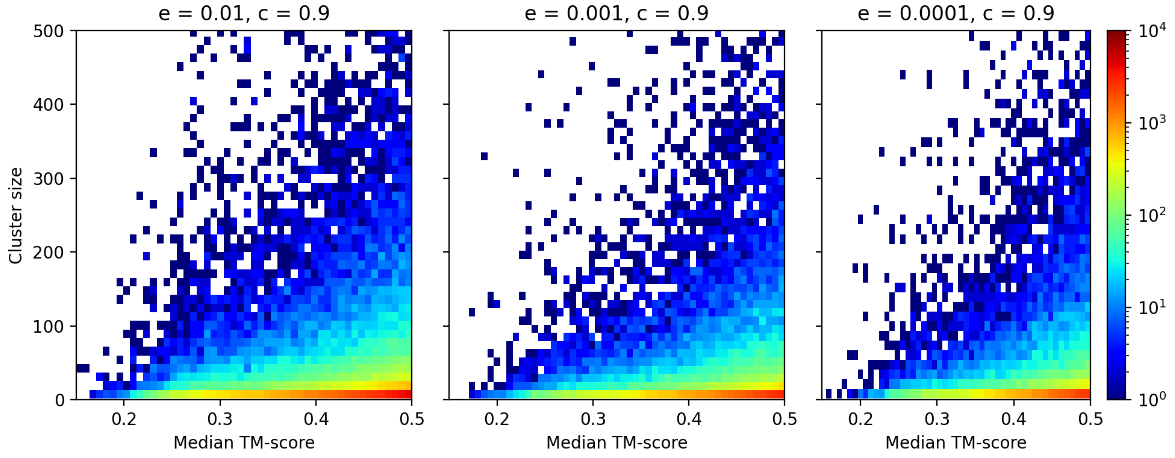

Supplementary Figure 13: Heatmap between median TM-score (chosen between mean TM-scores of cluster representative to all the other structures in a given cluster) and cluster size. Each panel corresponds to a different e-value, e, and coverage parameter, c, combination. In contrast to Supplementary Figure 12 we focused on small and medium clusters (between 2 and 500 elements) with poor quality (TM-score < 0.5).

Supplementary Table 1: Number of very poor large clusters (size > 500, median TM-score between representative structure and all the other structures per cluster < 0.4). Coverage parameter c = 0.9.

**E-value**	0.01	0.001	0.0001
**Number of clusters**	71	44	29

Taking all the above into consideration (especially the best cluster quality among all combinations and the largest number of cluster representatives), we chose **e-value = 0.0001 and c = 0.9** as the optimal clustering parameters.

Second stage: final dataset

Finally, to remove structural redundancy among different, already structurally clustered, datasets, we used Foldseek yet again but this time for coverage parameter c = 0.7, 0.8, and 0.9 (following the work of Barrio-Hernandez et al. or standards set by UniRef (1, 3)). Overall (except c = 0.9, e-value = 0.0001), the number of clusters and singletons (mean cluster size) increases (decreases) with e-value and coverage parameter, c – Supplementary Figure 14 and Supplementary Figure 15. Interestingly, the number of singletons for c = 0.9 is ~2 times larger as compared to c = 0.7 and 0.8.

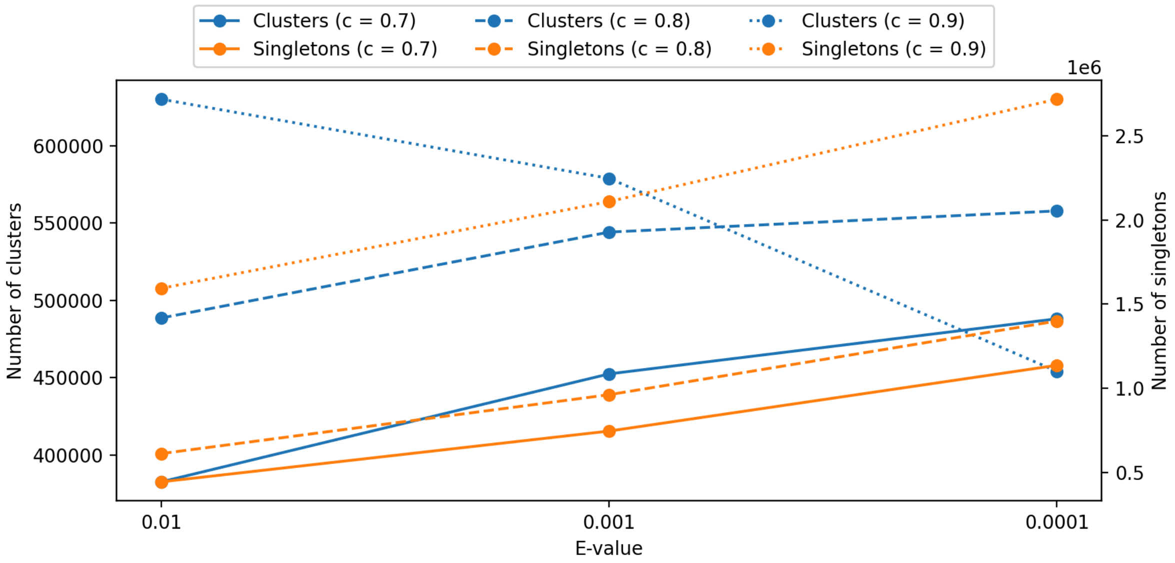

Supplementary Figure 14: Number of clusters (left y-axis) and singletons (right y-axis) as a function of e-value stratified by coverage parameter (c).

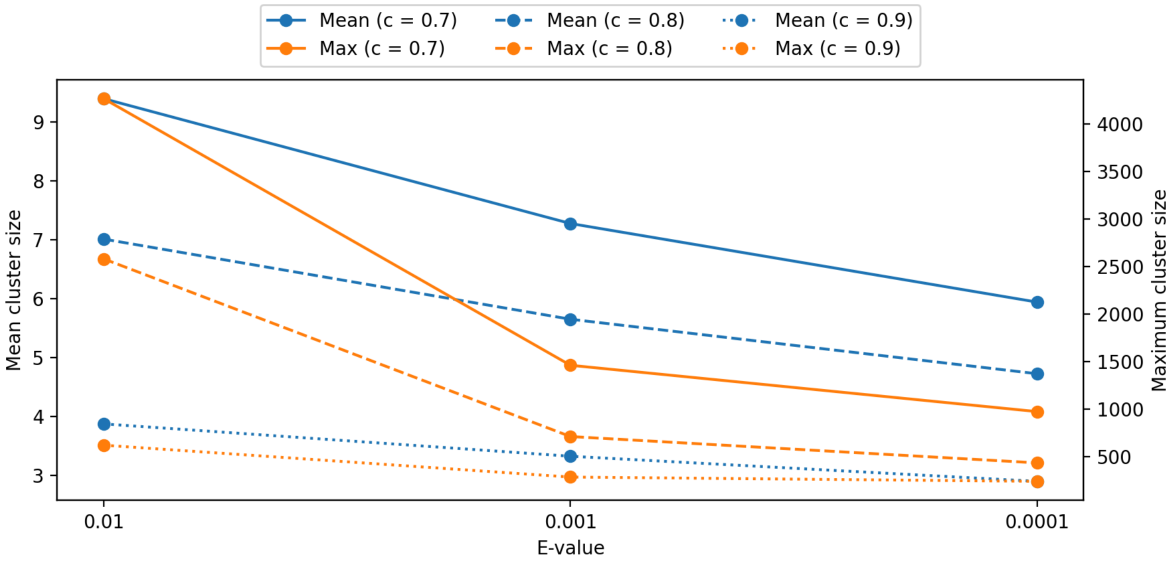

Supplementary Figure 15: Mean cluster size (left y-axis) and maximum cluster size (right y-axis) as a function of e-value stratified by coverage parameter (c). Note that the standard deviation is not meaningful here because the distribution of cluster sizes does not follow a normal distribution.

The TM-score distribution between the representative and all the other structures for the top 10 largest clusters shows that c = 0.7 is too permissive (see Supplementary Figure 16).

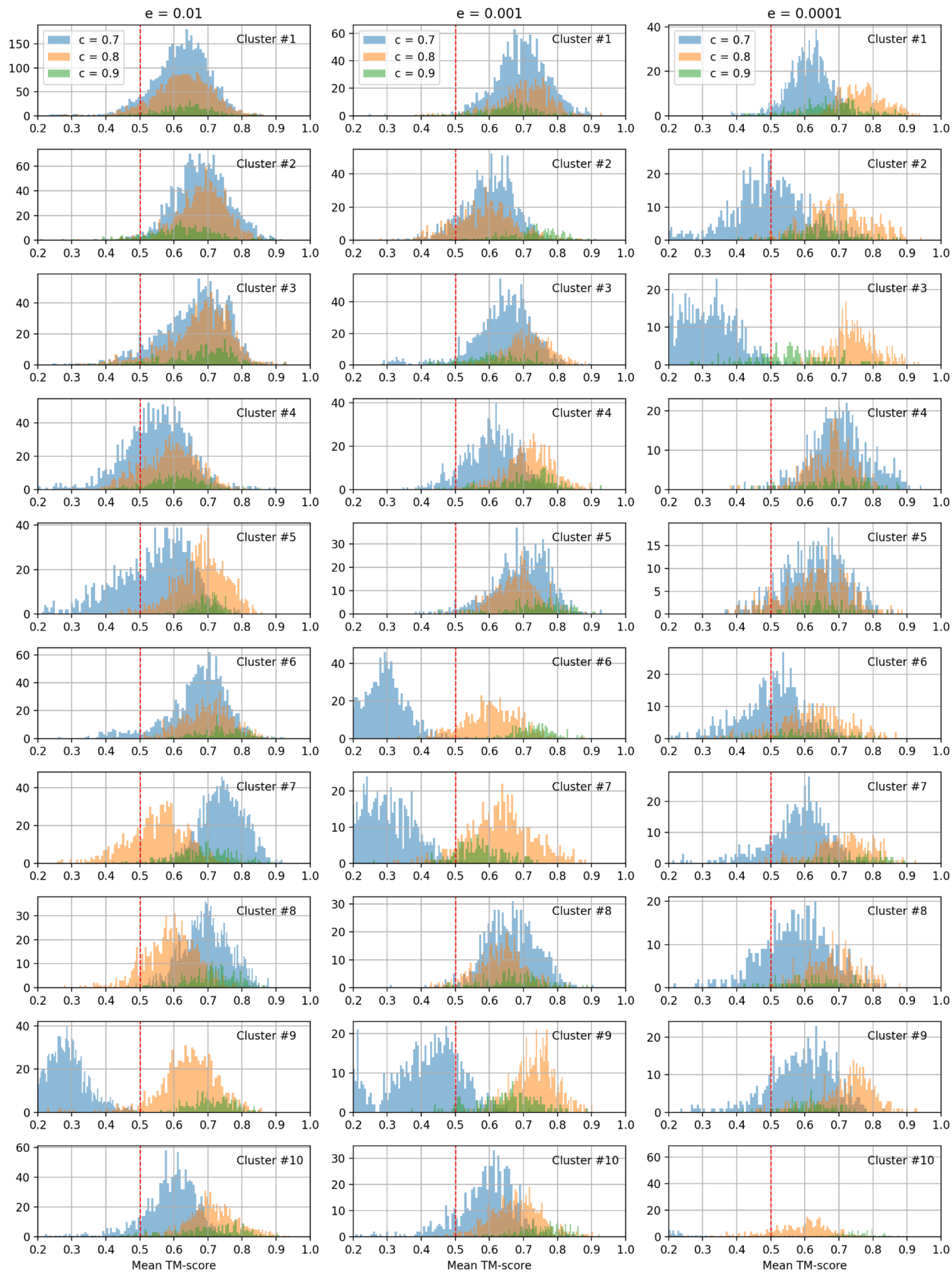

Supplementary Figure 16: Distribution of mean TM-score between each structure and representative structure (excluding identities) within a given cluster for the top 10 largest clusters. Each color corresponds to a different coverage parameter, c, whereas each column to a different e-value, e.

In summary, the optimal combination, providing a reasonable number of cluster representatives including singletons (reduction from 4,035,121 to 1,505,141) and high-quality clusters, is **e-value = 0.001 and c = 0.8**. Smaller/larger e-values would also give satisfactory results (contrary to the coverage parameter).

Supplementary Table 2 summarizes the number of structures in each clustered dataset (including final dataset). Note that we consider here all AFDB structures (including those with low pLDDT). Restricting to high-quality (pLDDT > 70) left us with ~62% light clusters and only 48% of dark clusters. Supplementary Figure 17 shows structure length distribution including only high-quality predictions. Supplementary Figure 18 presents distribution of mean pLDDT for AFDB structures from the full database (random samples) and clustered dataset. Clearly, we can notice a huge discrepancy between the two, caused by clustering procedures (many high-quality models are similar and have been removed due to redundancy).

Supplementary Table 2: Optimal Foldseek parameters, number of input structures, and the resulting number of clusters/singletons for each dataset (first stage clustering, first four rows). In yellow/red we indicate representative structures that have been used in/excluded from the second stage clustering (last row). In green we present final numbers of structures that are analyzed in this work. Number of input structures refers to non-redundant structures on a sequence level. Number of output structures refers to all structures gathered in the clusters (and singletons, but only for MIP and the final database). For the final database we show numbers of all output clusters, singletons, and structures as well as only the high-quality ones (i.e., with mean pLDDT > 70 for AFDB structures; other databases are already considered high-quality).

	Foldseek e-value	Foldseek coverage	Input structures	Clusters	Singletons	Output structures
**AFDB light***	0.01	0.9	52,327,413	1,591,199	13,012,338	30,045,210
**AFDB dark***				711,700		
**MIP**	0.001	0.9	211,069	17,628	83,986	211,069
**ESMAtlas**	0.0001	0.9	36,983,470	1,630,608	8,482,776	28,500,694
**Final database** **all**	0.001	0.8	4,035,121	544,123	961,018	4,035,121
**Final database** **hq**				422,003	648,398	3,060,808

*Clustering performed in reference (1).

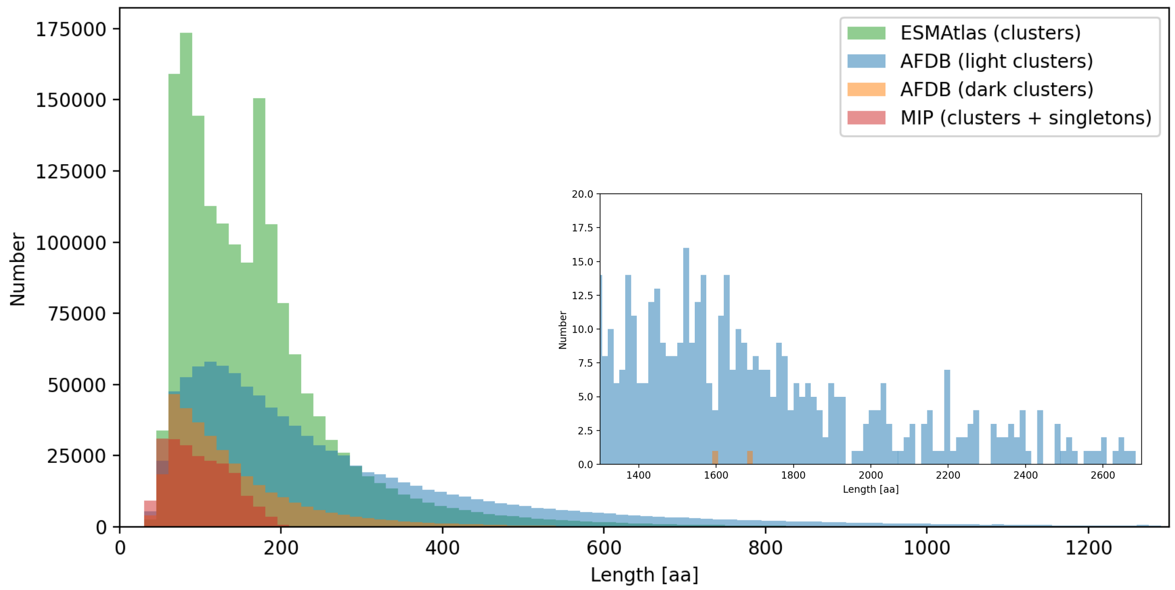

Supplementary Figure 17: Structure length distribution in the analyzed datasets (only high-quality predictions have been considered – see last row in Supplementary Table 2). Inset shows long proteins (not visible in the main panel).

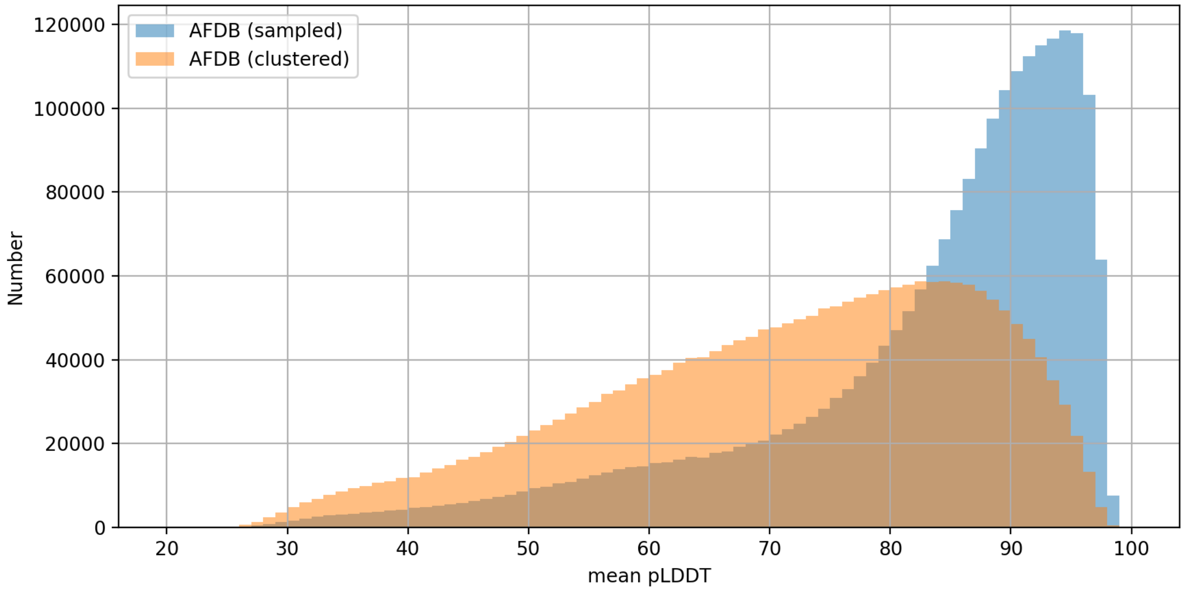

Supplementary Figure 18: Histogram of mean pLDDT for AFDB structures: randomly sampled from the entire database and coming from the clustered dataset. Both distributions have equal sample sizes for accurate comparison.

Structure space

Shape-mer representations generated with Geometricus for all representative structures in the final clustered database have been reduced into two dimensional space using PaCMAP (see Methods in the manuscript). We considered two versions of embedding vectors:

- unnormalized: raw shape-mer vector (summing up to the number of residues of a query protein)
- normalized: raw shape-mer vector divided by the sum of its elements (summing up to 1)

Of course, the second type is less biased by the structure length distribution so we chose it as a default.

PaCMAP grid search

In order to find optimal PaCMAP parameters we performed a grid search, changing:

- `n_neighbors` between 2 to 20 every 1
- `MN_ratio` between 0.1 to 5.1 every 0.2
- `FP_ratio` between 0.1 to 5.1 every 0.2

We wanted to ensure the best separation between clusters from Supplementary Figure 16 but 9 out of 10 cluster representatives are alpha-like and one is alpha/beta-like. Therefore, we decided to add 5 additional clusters that are beta- and alpha/beta-like (see Supplementary Figure 19). This yielded 8,180 structures, for which we used the agreement TM-score as a measure of similarity.

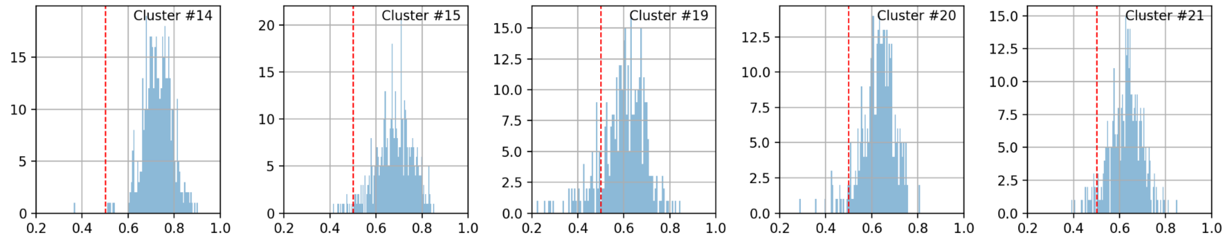

Supplementary Figure 19: The same as in Supplementary Figure 16 but for e-value = 0.001, c = 0.8 and for manually chosen beta- and alpha/beta-like clusters.

We wanted to preserve both global (between clusters) and local (within a cluster) separation. For each PacMAP reduction we computed correlation (Pearson and Spearman coefficients) between Euclidean distance for each pair of points (structures) and their mean TM-score, taking two inputs:

- representative structures – see Supplementary Figure 20
- all structures from each cluster (we take mean correlation coefficient as a final outcome) – see Supplementary Figure 21 and Supplementary Figure 22

Our maximization function is simply a product of the two coefficients defined above, separately for Pearson and Spearman – note that both are negative so their product is positive. Supplementary Figure 23 gathers the results.

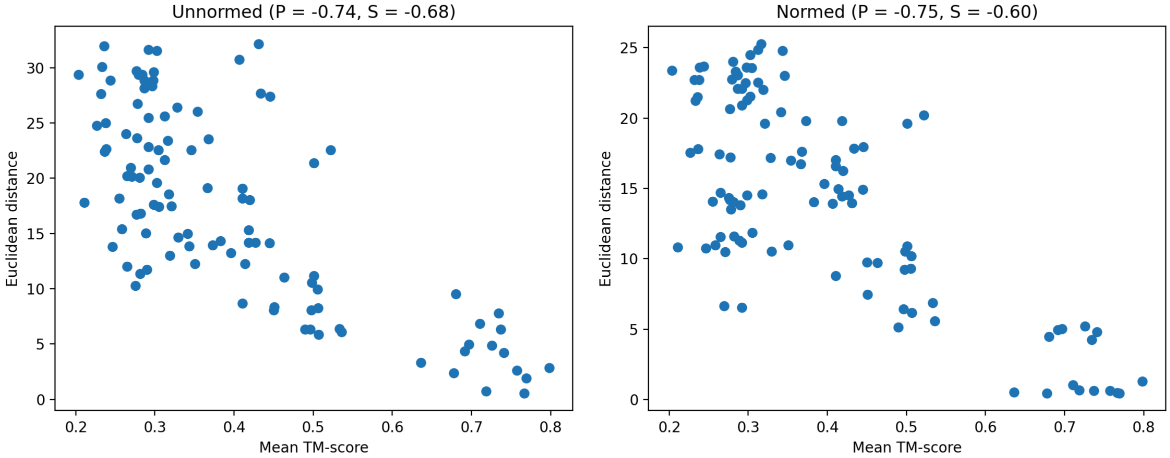

Supplementary Figure 20: Mean TM-score versus Euclidean distance for representative structures. Left/right: unnormalized/normalized shape-mer representation. In the title P and S denote Pearson and Spearman correlation coefficients respectively.

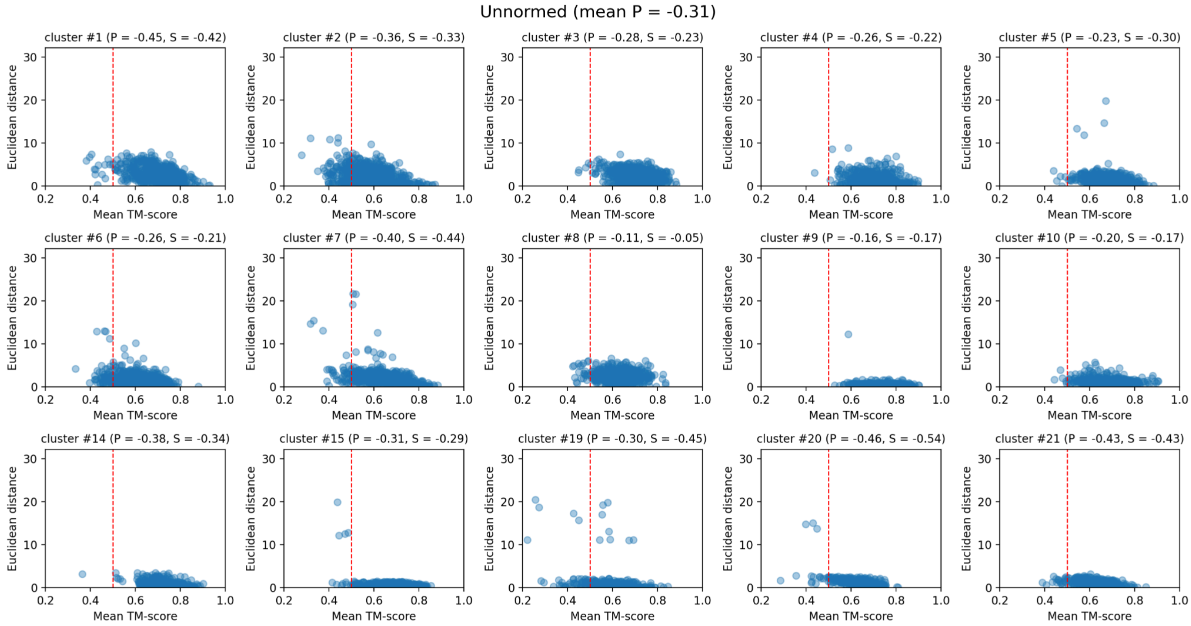

Supplementary Figure 21: Mean TM-score versus Euclidean distance for all structures within a given cluster for unnormalized shape-mer representation. In the titles P and S denote Pearson and Spearman correlation coefficients respectively.

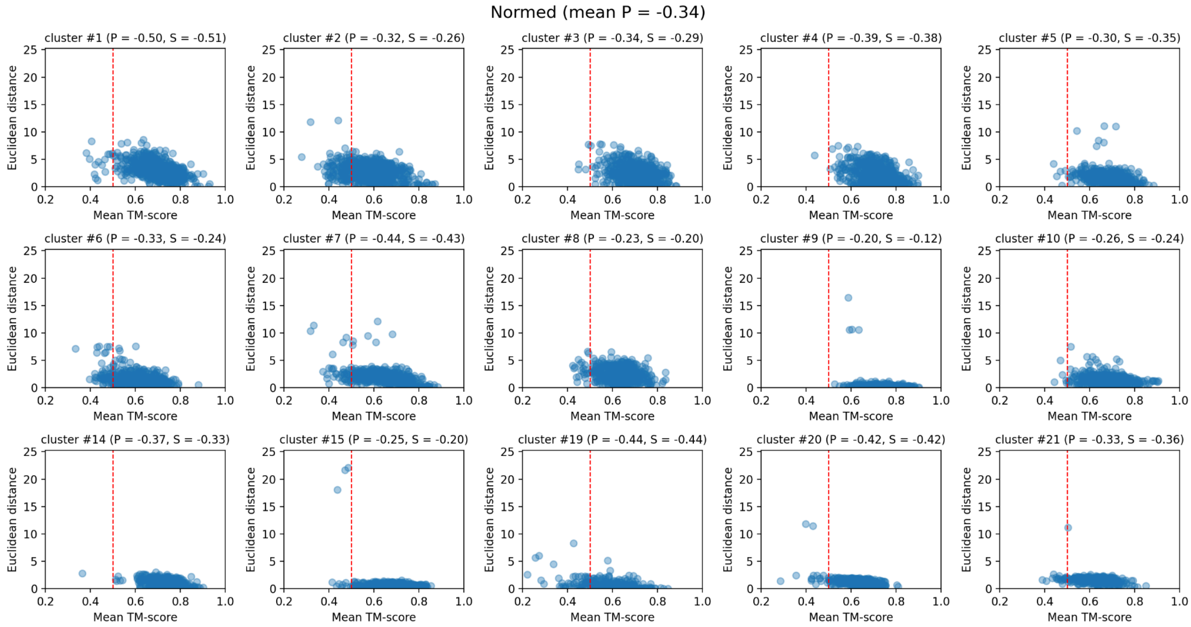

Supplementary Figure 22: Mean TM-score versus Euclidean distance for all structures within a given cluster for normalized shape-mer representation. In the titles P and S denote Pearson and Spearman correlation coefficients respectively.

There are a few comment worth noting:

- In general, larger Spearman and Pearson products are obtained for normalized Geometricus representations (panel a in Supplementary Figure 23).
- We can observe certain relations between `MN_ratio` and `FP_ratio`, and default parameters may not always be optimal (panel b in Supplementary Figure 23).
- Default value of `n_neighbors` seems to be quite robust and in our case any value equal or larger than 10 gives good performance (panel c in Supplementary Figure 23).

In summary, the following sets of optimal PaCMAP parameters (`n_neighbors`, `MN_ratio`, `FP_ratio`) has been chosen:

- unnormalized: (13, 1.9, 1.5)
- normalized: (10, 1.3, 0.9)

Supplementary Figure 24 and Supplementary Figure 25 illustrate the PaCMAP reduction in action, showing a clear separation between alpha and beta clusters. Notably, within the alpha clusters, which overrepresent the top largest clusters, there is a distinct arrangement reflecting finer details of the folds. Obviously, for the unnormalized Geometricus vectors, proteins are distributed according to structure length, a pattern not observed with the normalized inputs.

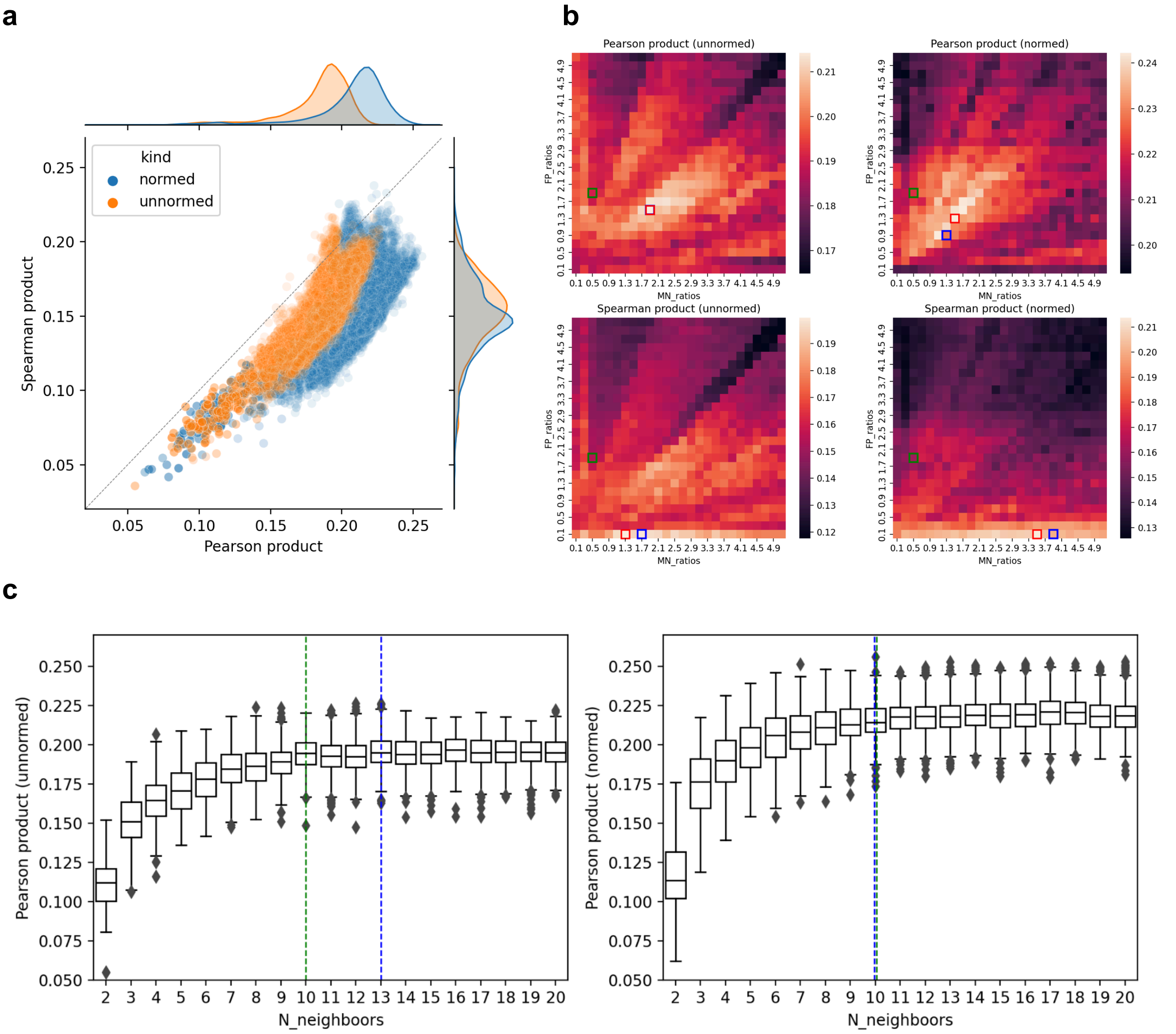

Supplementary Figure 23: Summary of PaCMAP grid search. (a): Scatter plot of Pearson and Spearman products (see the text for definitions) for two versions of shape-mer representations (normalized and unnormalized). **(b)**: Median values (for `n_neighbors`) of Pearson and Spearman products for each tested combination of `MN_ratio` and `FP_ratio` parameters. Green/blue/red rectangles on each panel represent default/optimal for specific `n_neighbors` (the best in this case)/optimal for all `n_neighbors` (the most robust) combinations respectively. **(c)**: Dependance between Pearson and Spearman products and `n_neighbors` parameter (each box has been generated using outcomes for all `MN_ratio` and `FP_ration` parameters). Green/blue dashed lines represent default/optimal for specific `MN_ratio` and `FP_ration` (the best in this case) values respectively. Data are presented as median values with interquartile range (IQR); whiskers indicate variability outside the upper and lower quartiles, and individual points represent outliers. Number of data points is equal to 650 for `n_neighbors` between 2 and 5, and equal to 676 for `n_neighbors` between 6 and 20.

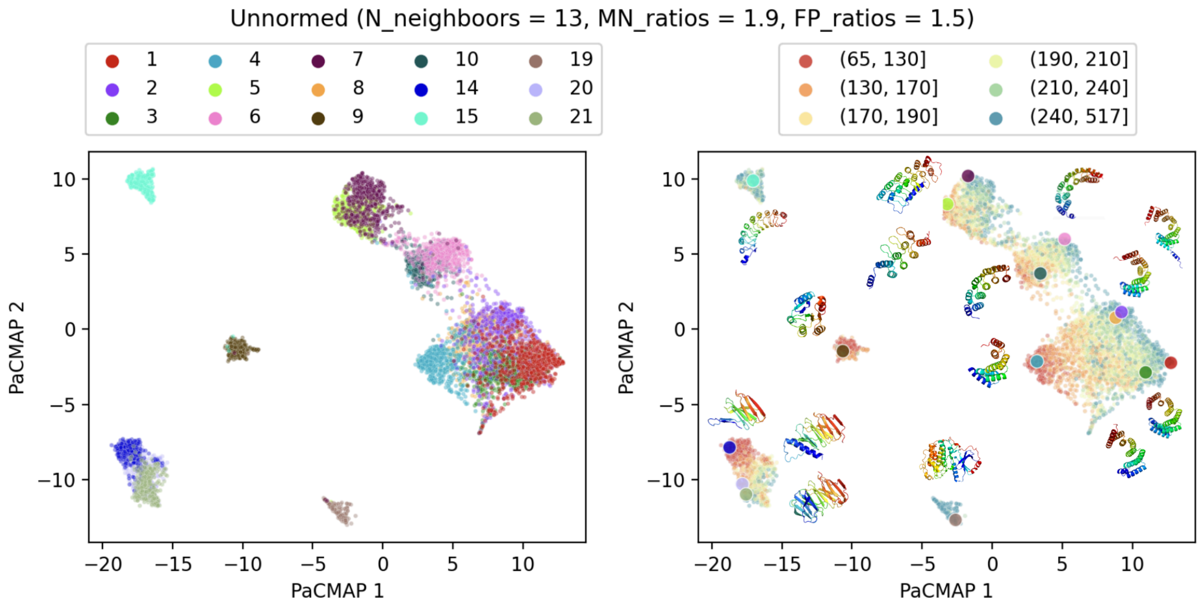

Supplementary Figure 24: Visualization of the protein structure space using PaCMAP (optimal parameters have been used) and unnormalized Geometricus representations for the top 15 largest clusters (see Supplementary Figure 16 and Supplementary Figure 19). Left: cluster index. Right: structure length as number of residues (see legend) and PyMOL depictions of representative structure in each cluster.

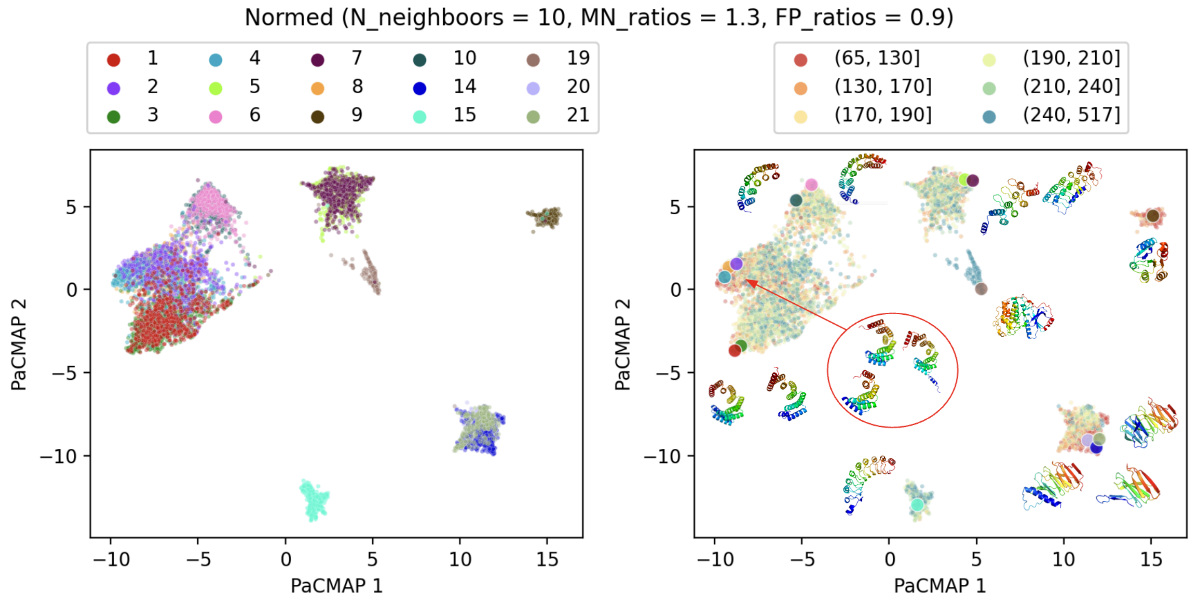

Supplementary Figure 25: As in Supplementary Figure 24 but for normalized Geometricus representations.

Visualizations

Here, we provide additional panels (supplementary to the main text) for visualizing 2D protein structure space using PaCMAP, for both normalized and unnormalized Geometricus representations.

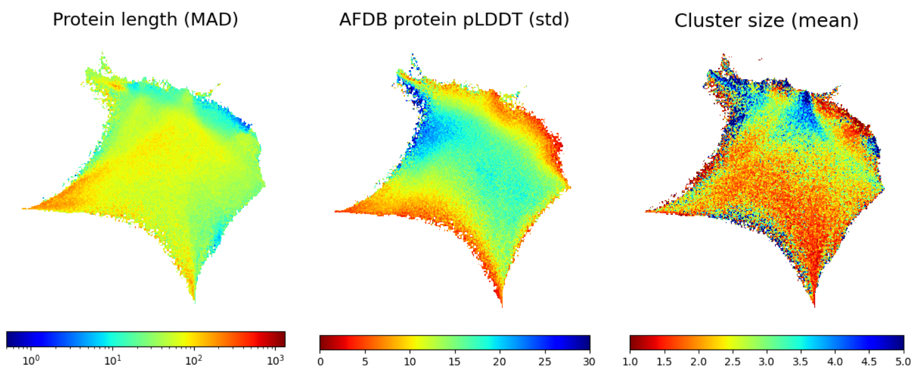

Supplementary Figure 26: Dispersion (MAD: median absolute deviation/std: standard deviation) of protein length and AFDB pLDDT as well as mean cluster size for representative structures (compare with Fig. 1b in the manuscript).

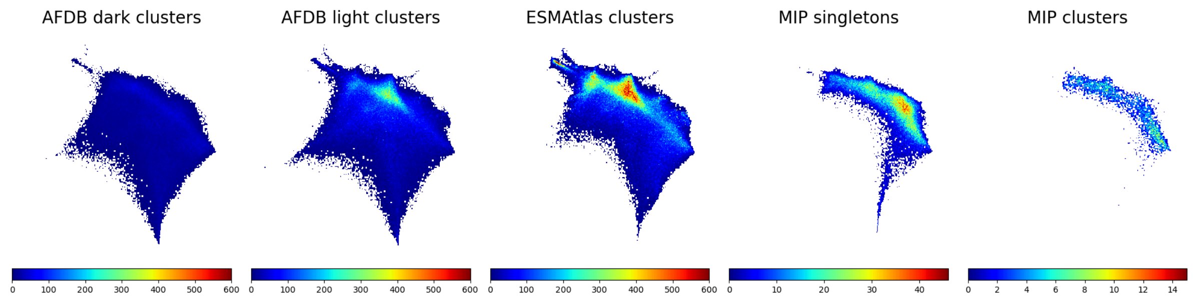

Supplementary Figure 27: The same as in Fig. 1c in the manuscript but the total number of structures (absolute values) is shown.

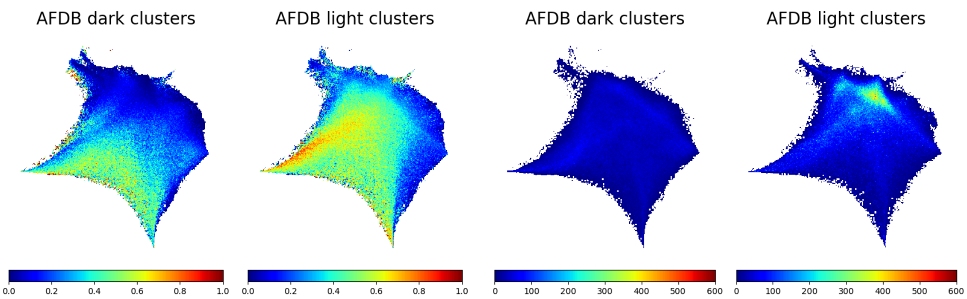

Supplementary Figure 28: The same as in Fig. 1c in the manuscript and Supplementary Figure 27 but for all AFDB models (two left plots: coverage, two right plots: total number of structures).

Plots for unnormalized Geometricus representations

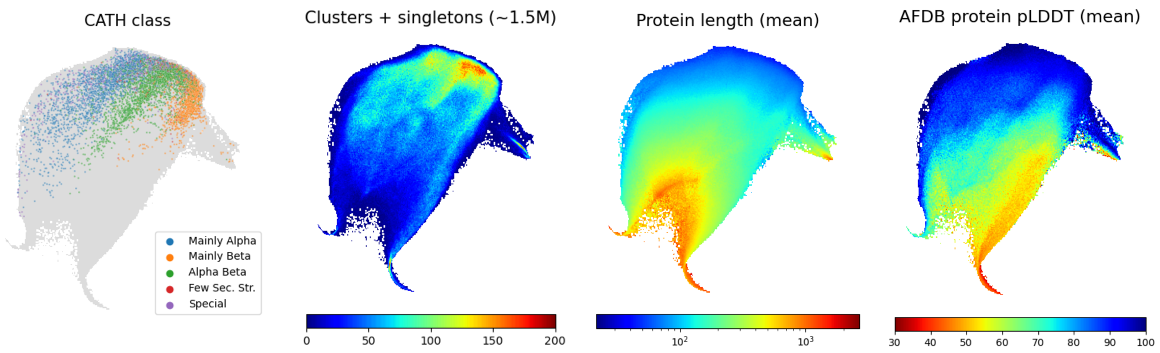

Supplementary Figure 29: The same as in Fig. 1b but for unnormalized Geometricus representations.

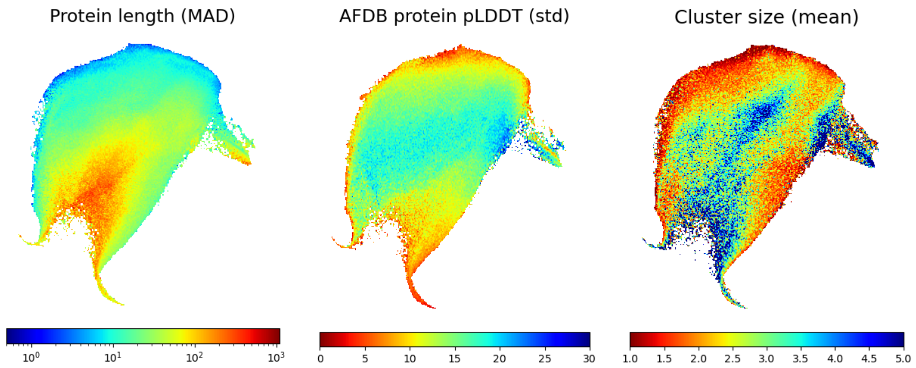

Supplementary Figure 30: The same as in Supplementary Figure 26 but for unnormalized Geometricus representations.

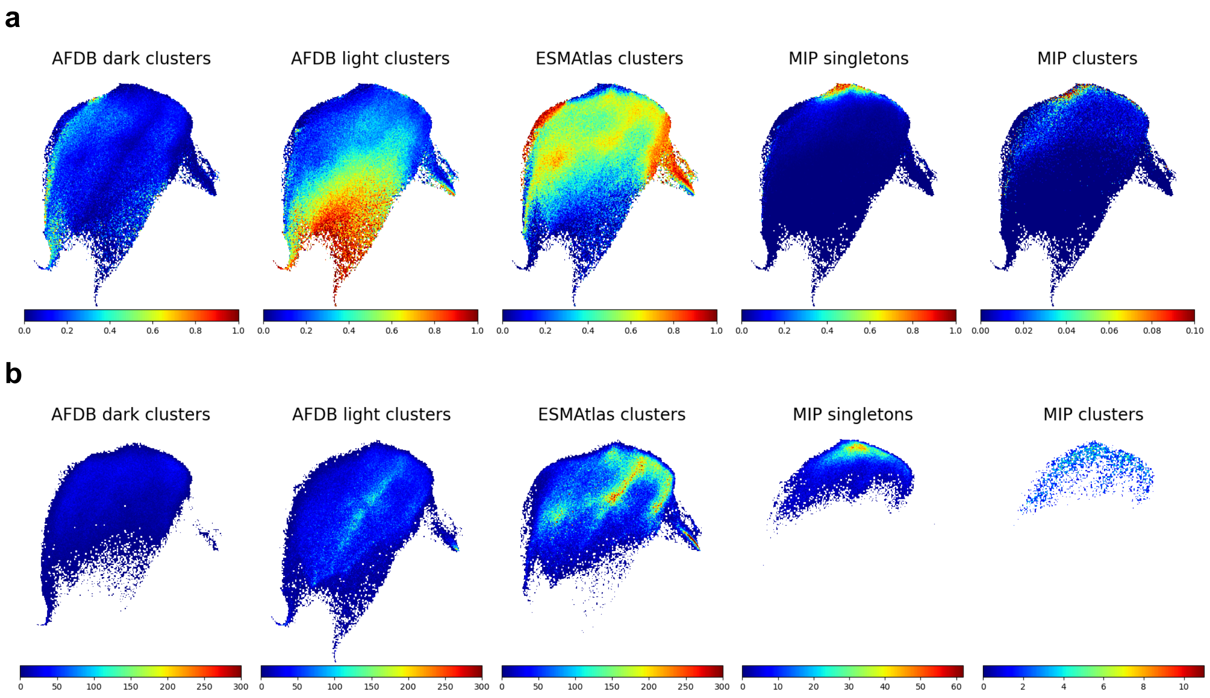

Supplementary Figure 31: (a): The same as in Fig. 1c in the manuscript but for unnormalized Geometricus representations. **(b):** Total number of structures (absolute values) is shown – compare with Supplementary Figure 27.

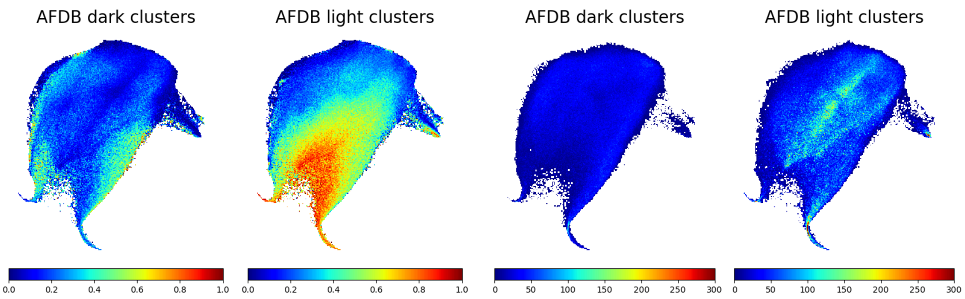

Supplementary Figure 32: The same as in Supplementary Figure 28 but for unnormalized Geometricus representations.

Other databases

To assess the generalizability of our dimensionality reduction approach, we evaluated it on two additional datasets: a smaller set of 10,000 AlphaFold2 models for sequences generated with ProtGPT2, and a larger set of 351,242 structural models from the Big Fantastic Viral Database (BFVD). For each structure in these datasets, we first computed Geometricus representations. Dimensionality reduction was then performed independently for each dataset (ProtGPT2 and BFVD) using PaCMAP, employing two distinct strategies:

1. Transformation using a pre-existing mapping – based on the AFDB, ESMAtlas, and MIP datasets – via the `transform()` method.
2. Fitting and transformation using an extended mapping – combining the AFDB, ESMAtlas, MIP, and the new dataset (ProtGPT2 or BFVD) – via the `fit_transform()` method.

The first approach is particularly useful when the goal is to project new structures into an existing embedding space – such as when identifying structural neighbors – though it's important to note that shapemers absent from the original Geometricus vocabulary are replaced with zeros. The second approach is more suitable when the objective is to understand how new datasets reshape or expand the existing structural landscape.

Supplementary Figure 33 and Supplementary Figure 34 illustrate the results of this analysis. For the ProtGPT2 dataset, proteins are distributed fairly evenly across the structural landscape, and minimal differences are observed between the two embedding strategies. In contrast, the BFVD dataset exhibits a more pronounced divergence between the two approaches. Using the transformation-only method (left panel of Supplementary Figure 34), the data reveals a concentration of alpha-helical proteins (visible in the lower corner). Viral proteomes comprise small single-helical proteins that, despite their structural simplicity, perform diverse critical functions during viral infection cycles. These compact elements contribute to viral assembly, membrane manipulation, immune antagonism, and host cellular pathway modulation. However, after fitting the embedding to include also the BFVD structures (right panel of Supplementary Figure 34) several notable changes become apparent:

1. Emergence of small, distant clusters – these outlier regions, labeled A–D, consist of short, low-complexity proteins.
2. Expansion of low-confidence region – the lower-left area, typically populated by low pLDDT structures, expands further due to the inclusion of additional low-complexity models (points E–H).
3. Displacement of alpha-helical cluster – the previously dense alpha-helical region is pushed outward from the main landscape (see area around point J).
4. Enrichment of a distinct region – a unique area at the top of the embedding space (surrounding point I) becomes enriched with BFVD proteins, predominantly long (>2000 amino acids) replicases and RNA-directed RNA polymerases.

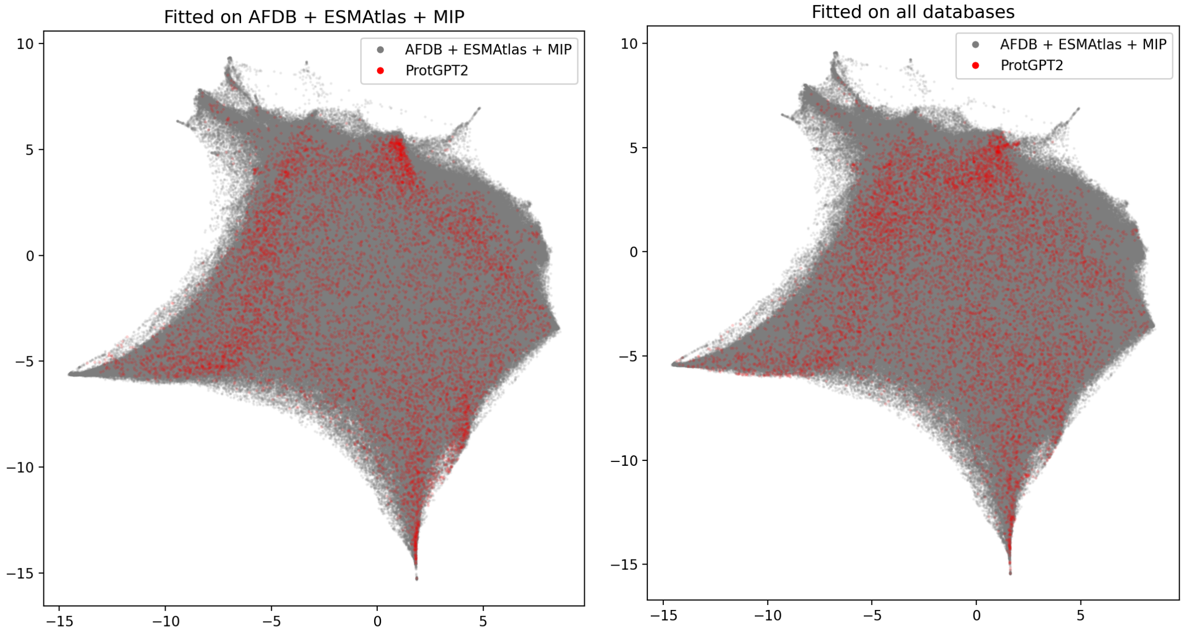

Supplementary Figure 33: 2-dimensional PaCMAP representation of 10,000 ProtGPT2-generated proteins, transformed using a PaCMAP model trained on AFDB, ESMAtlas, and MIP representative structures (left panel), as well as on all databases, including ProtGPT2 (right panel).

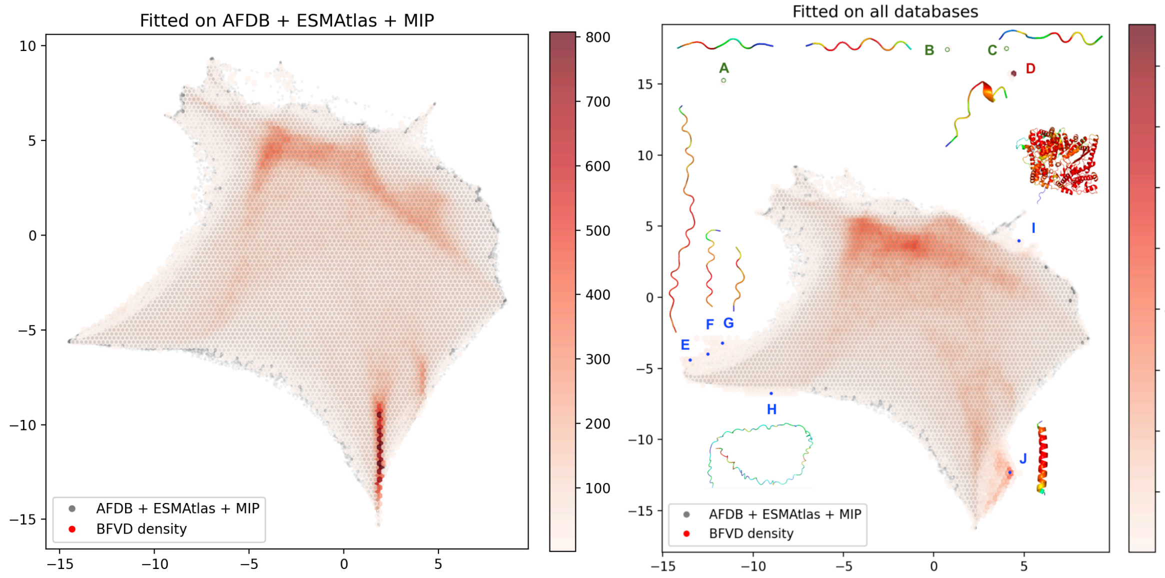

Supplementary Figure 34: 2-dimensional PaCMAP representation of 351,242 BFVD proteins, transformed using a PaCMAP model trained on AFDB, ESMAtlas, and MIP representative structures (left panel), as well as on all databases, including BFVD (right panel). Outlier points at the top of the right panel (labels: A, B, C) are highlighted with green circles for better visibility. Names of structural models in the right panel (labels: A-J) are shown in Supplementary Table 3.

Supplementary Table 3: Names of structural models presented in the right panel of Supplementary Figure 34.

Point	Name
A	Q9IBY9_unrelaxed_rank_001_alphafold2_ptm_model_1_seed_000
B	M1HRI5_unrelaxed_rank_001_alphafold2_ptm_model_1_seed_000

C	F1ATE0_unrelaxed_rank_001_alphafold2_ptm_model_3_seed_000
D	H9CID8_unrelaxed_rank_001_alphafold2_ptm_model_3_seed_000
E	L7TJB1_unrelaxed_rank_001_alphafold2_ptm_model_2_seed_000
F	M1PSG3_unrelaxed_rank_001_alphafold2_ptm_model_3_seed_000
G	M9V8N1_unrelaxed_rank_001_alphafold2_ptm_model_3_seed_000
H	A0A0F7L506_unrelaxed_rank_001_alphafold2_ptm_model_3_seed_000
I	A0A2P1GNB0_1_unrelaxed_rank_001_alphafold2_ptm_model_3_seed_000
J	A0A8S5P478_unrelaxed_rank_001_alphafold2_ptm_model_1_seed_000

To estimate how distinct the ProtGPT2 and BFVD datasets are from those used in our study, we applied foldseek easy-cluster to the original dataset of ~4 million proteins (prior to stage 2), supplemented with either 10,000 randomly generated structures from ProtGPT2 or 351,242 structures from BFVD. Clustering was performed using two parameter sets: (e-value = 0.01, coverage = 0.7) and (e-value = 0.001, coverage = 0.8). In both ProtGPT2 and BFVD, we observed a substantial number of singleton clusters, suggesting a high degree of structural uniqueness (see Supplementary Table 4). For BFVD, this outcome was anticipated, given that viral proteins are scarcely represented in other databases (approximately 2% of the AFDB). To further explore the uniqueness of ProtGPT2-generated structures, we conducted foldseek easy-search against the AFDB, ESMAtlas, and MIP representative datasets using the `--greedybest-hits` option. The proportion of proteins with no detectable homologs was consistent with the clustering analysis: 73.3% at e-value = 0.001 and 69.6% at e-value = 0.01. To understand this lack of matches, we analyzed the mean pLDDT and pTM scores of the ProtGPT2 structures. Singletons exhibited significantly lower AlphaFold confidence scores, which may account for the absence of detectable homologs (see Supplementary Figure 35). Random selection of singleton and non-singleton ProtGPT2 models is presented in Supplementary Figure 37 (see also Supplementary Table 5). The distribution of mean pLDDT scores in the BFVD dataset was more uniform. Notably, we identified a subset of high-confidence structures (mean pLDDT between 80 and 90), many of which correspond to alpha-helical proteins described above (see Supplementary Figure 36).

Supplementary Table 4: Percentage of ProtGPT2 (first row) and BFVD (second row) singletons after the second stage clustering with foldseek easy-cluster. In both cases all 4,035,121 representative proteins from clustered AFDB, clustered ESMAtlas and MIP databases have been used (see Supplementary Table 2).

	**e-value = 0.01 coverage = 0.7**	**e-value = 0.001 coverage = 0.8**
**ProtGPT2**	62.2%	71.6%
**BFVD**	53.9%	64.1%

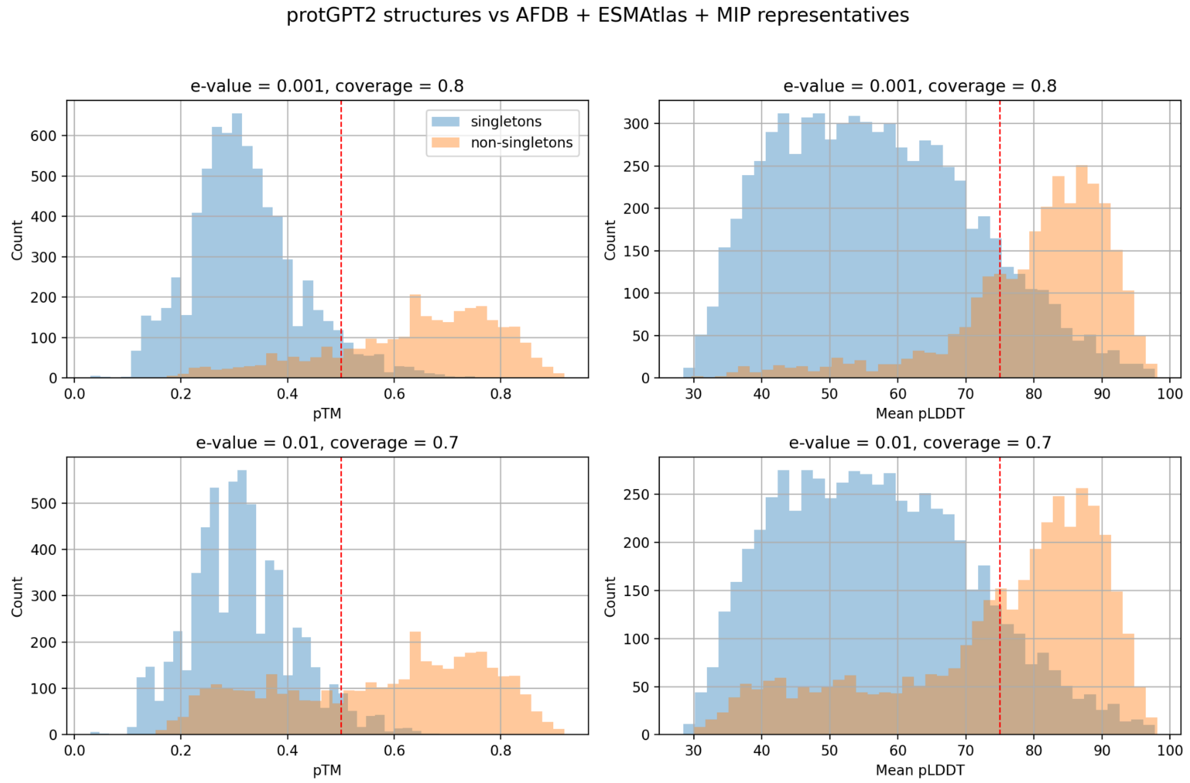

Supplementary Figure 35: pTM and mean pLDDT scores (left and right column respectively) for 10,000 ProtGPT2 protein structures without homologs (singletons after foldseek clustering) and with homologs (non-singletons). We used two sets of easy-cluster parameters (upper and lower panels respectively).

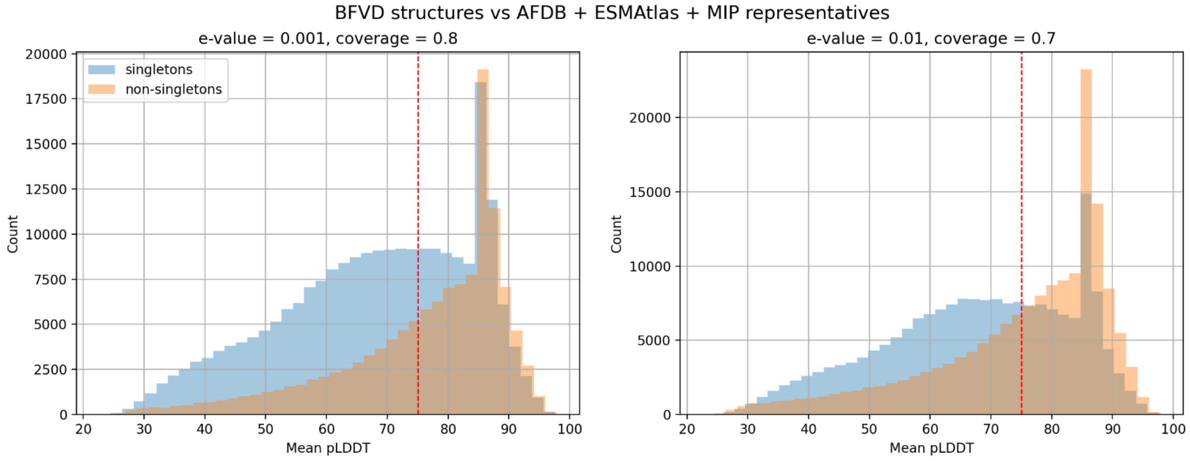

Supplementary Figure 36: Mean pLDDT for 351,242 protein structures without homologs (singletons after foldseek clustering) and with homologs (non-singletons). We used two sets of easy-cluster parameters (left and right panel respectively).

a

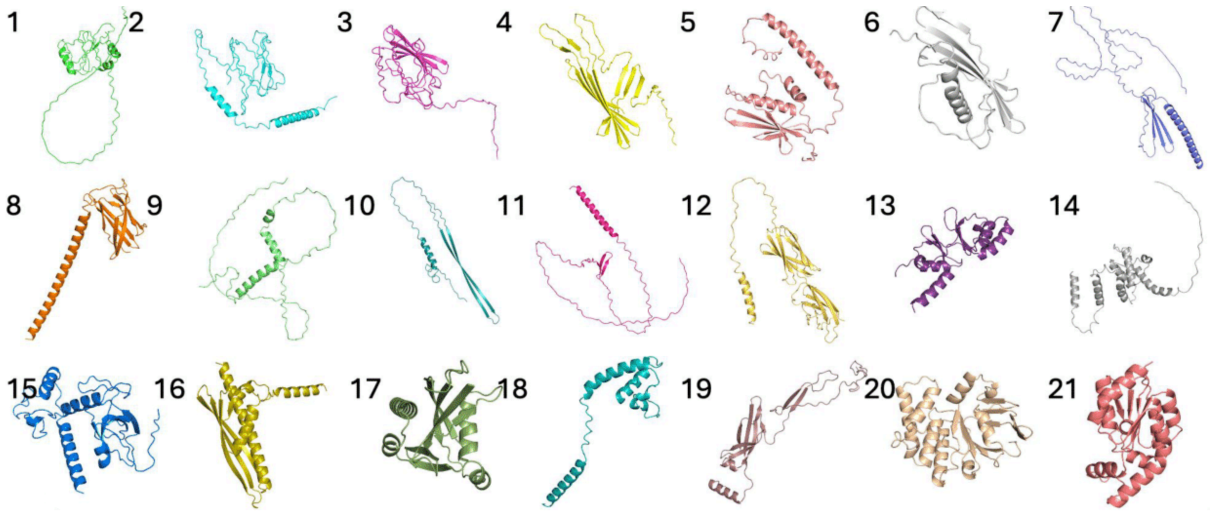

b

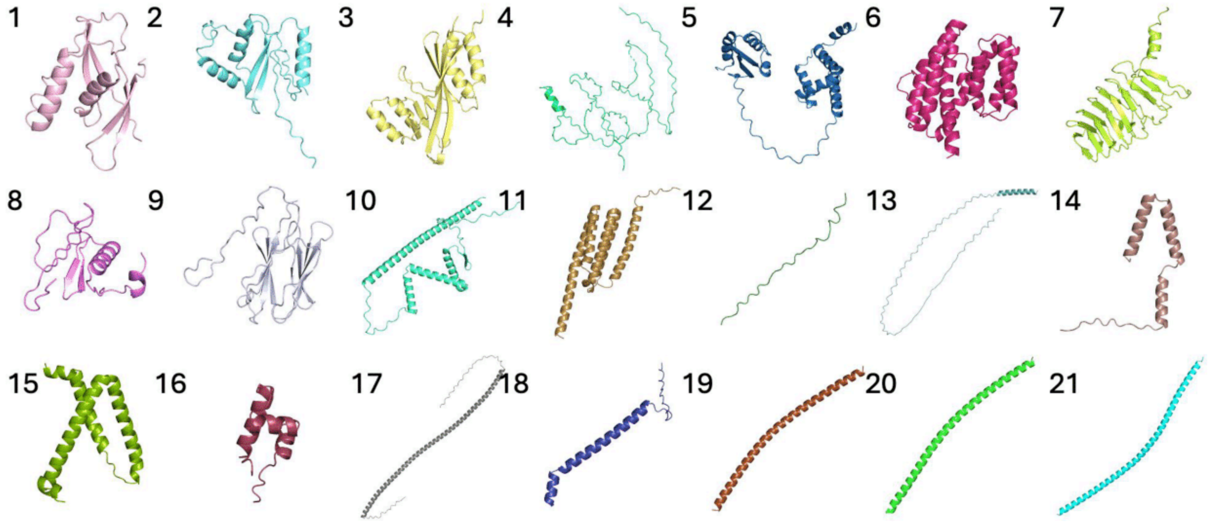

Supplementary Figure 37: Random selection of ProtGTP2 models (see Supplementary Table 5), stratified by pLDDT range and cluster membership with 3 models per pLDDT scoring range. **(a)** Non-singletons and **(b)** singletons obtained from foldseek clustering with e-value = 0.001 and coverage = 0.8. Each group is subdivided into seven pLDDT intervals (30–40 to 90–100), enabling visual comparison of fold quality and diversity as a function of structural confidence and clusterability.

Supplementary Table 5: Details of the models presented in Supplementary Figure 37.

#	cluster	pLDDT range	model name
a1	non-singleton	30-40	3999_unrelaxed_rank_1_model_2.pdb
a2	non-singleton	30-40	6198_unrelaxed_rank_1_model_2.pdb
a3	non-singleton	30-40	6879_unrelaxed_rank_1_model_2.pdb
a4	non-singleton	40-50	3905_unrelaxed_rank_1_model_4.pdb
a5	non-singleton	40-50	5860_unrelaxed_rank_1_model_5.pdb
a6	non-singleton	40-50	6651_unrelaxed_rank_1_model_1.pdb

a7	non-singleton	50-60	4724_unrelaxed_rank_1_model_4.pdb
a8	non-singleton	50-60	7686_unrelaxed_rank_1_model_2.pdb
a9	non-singleton	50-60	8518_unrelaxed_rank_1_model_3.pdb
a10	non-singleton	60-70	2402_unrelaxed_rank_1_model_2.pdb
a11	non-singleton	60-70	2477_unrelaxed_rank_1_model_2.pdb
a12	non-singleton	60-70	3075_unrelaxed_rank_1_model_2.pdb
a13	non-singleton	70-80	2048_unrelaxed_rank_1_model_4.pdb
a14	non-singleton	70-80	2955_unrelaxed_rank_1_model_1.pdb
a15	non-singleton	70-80	3663_unrelaxed_rank_1_model_3.pdb
a16	non-singleton	80-90	1492_unrelaxed_rank_1_model_3.pdb
a17	non-singleton	80-90	1952_unrelaxed_rank_1_model_4.pdb
a18	non-singleton	80-90	586_unrelaxed_rank_1_model_3.pdb
a19	non-singleton	90-100	144_unrelaxed_rank_1_model_4.pdb
a20	non-singleton	90-100	206_unrelaxed_rank_1_model_5.pdb
a21	non-singleton	90-100	347_unrelaxed_rank_1_model_5.pdb
b1	singleton	30-40	6488_unrelaxed_rank_1_model_2.pdb
b2	singleton	30-40	7481_unrelaxed_rank_1_model_4.pdb
b3	singleton	30-40	9680_unrelaxed_rank_1_model_4.pdb
b4	singleton	40-50	3579_unrelaxed_rank_1_model_2.pdb
b5	singleton	40-50	4459_unrelaxed_rank_1_model_2.pdb
b6	singleton	40-50	6773_unrelaxed_rank_1_model_4.pdb
b7	singleton	50-60	4988_unrelaxed_rank_1_model_4.pdb
b8	singleton	50-60	7284_unrelaxed_rank_1_model_2.pdb
b9	singleton	50-60	8813_unrelaxed_rank_1_model_2.pdb
b10	singleton	60-70	3285_unrelaxed_rank_1_model_3.pdb
b11	singleton	60-70	4991_unrelaxed_rank_1_model_2.pdb
b12	singleton	60-70	9618_unrelaxed_rank_1_model_4.pdb
b13	singleton	70-80	3059_unrelaxed_rank_1_model_2.pdb
b14	singleton	70-80	6561_unrelaxed_rank_1_model_5.pdb
b15	singleton	70-80	9646_unrelaxed_rank_1_model_3.pdb
b16	singleton	80-90	198_unrelaxed_rank_1_model_3.pdb
b17	singleton	80-90	3674_unrelaxed_rank_1_model_3.pdb
b18	singleton	80-90	6062_unrelaxed_rank_1_model_5.pdb
b19	singleton	90-100	2837_unrelaxed_rank_1_model_3.pdb
b20	singleton	90-100	3281_unrelaxed_rank_1_model_5.pdb
b21	singleton	90-100	3669_unrelaxed_rank_1_model_3.pdb

Functional annotations

deepFRI v1.1

For training deepFRI v1.1 we utilized structures from the AlphaFold database v4 (https://alphafold.ebi.ac.uk/) and functional annotations from the Gene Ontology Annotation (GOA) Database (https://geneontology.org/). First, we selected GO terms with at least one experimental annotation and a total of 50 to 5000 annotations (excluding IEA). Additionally, we chose structures with ≥ 80% residues having pLDDT ≥ 70, and size between 60 and 1000 residues. This produced 2,822,622 structures annotated by 6,530 GO-terms and 4,905,062 annotations in total. Next, we enriched annotations by propagating GO-terms upwards in the GO-graph. This means that if a structure is annotated with a specific GO-term, it is also automatically annotated with all more general GO-terms linked to that term. This resulted in 8,877 GO-terms and 59,731,892 annotations in total. Lastly, the set has been randomly split into training and validation sets with the same proportions as in the original deepFRI release and three separate neural networks (for CC, MF and BP) have been trained with early stopping. Calibration curves showing precision versus deepFRI score (indicating how the method is certain about its predictions) for deepFRI v1.0 testing set are shown in Supplementary Figure 38. For both versions precision increases with the score but, at least for structure from the PDB, we should consider a higher deepFRI score for v1.1 as compared to v.1.0 in order to obtain similar quality predictions (e.g. 0.5 and 0.35 respectively for precision ≥ 0.5). We speculate that this may be caused by the fact that v1.1 has been trained with a much larger average number of structures per GO-term. Even though v1.1 produces more false positives for a given score, it predicts vastly more GO-terms as compared to v1.0 and may be especially useful for novel fold annotations (but manual curation is desired in such cases).

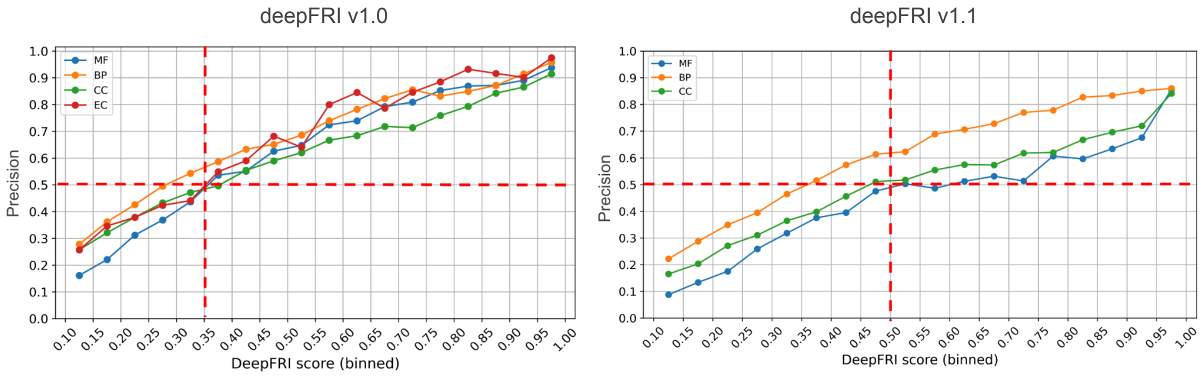

Supplementary Figure 38: Relation between precision and binned deepFRI score for releases 1.0 and 1.1 stratified by ontology (model), using deepFRI v1.0 testing set. The testing set comprises structures from the Protein Data Bank (PDB) with sequence similarity ≤ 30% to deepFRI v1.0 training set (see reference (4) for details).

Validation using E. coli proteome

We compared *E. coli* K12 proteome annotations derived from deepFRI v1.0, v1.1 with validated Swissprot records to benchmark our score thresholds – see Supplementary Figure 39. In general, deepFRI v1.0 is more accurate at scores ≥ 0.3, whereas deepFRI v1.1 shows higher concordance with Swissprot records at scores ≥ 0.5. However, annotations

associated with superCOG 3 are less accurate in deepFRI v1.1 when compared to deepFRI v1.0.

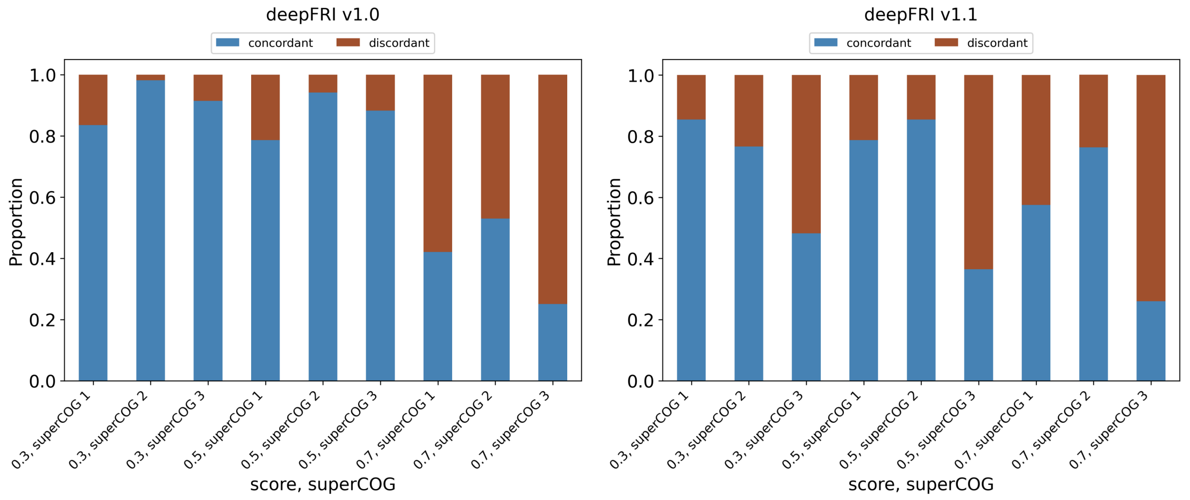

Supplementary Figure 39: Concordance and discordance plots (with respect to the Swissprot records) on superCOG level for *E. coli* K12 proteome. Benchmarks for deepFRI v 1.0 and v1.1 are shown for different score thresholds (0.3, 0.5 and 0.7).

Supplementary Figure 40 presents the aggregation of superCOG annotations for both deepFRI versions, Swissprot records, and the COG classifier (https://github.com/moshi4/COGclassifier).

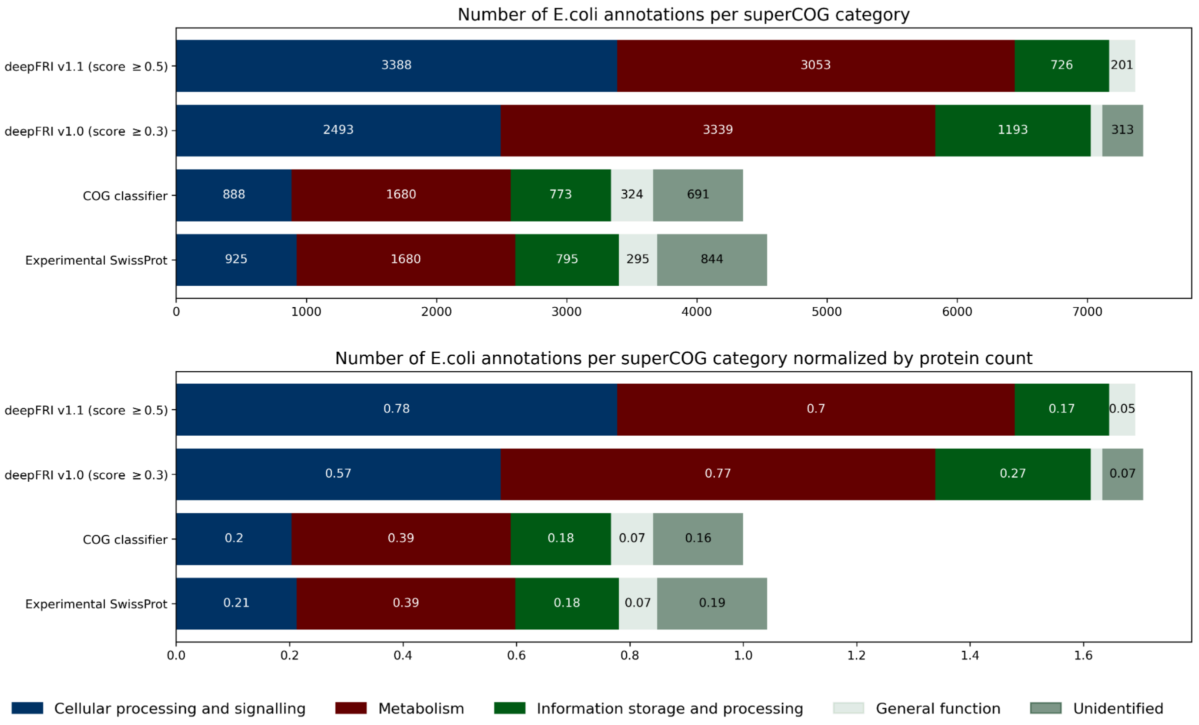

Supplementary Figure 40: Comparative analysis of *E. coli* K12 proteome annotations using deepFRI and COG classifier to Swissprot experimental records. Upper panel: total number of annotations. Lower panel: total number of annotations divided by the number of proteins. In the latter case, given the redundancy of annotations, more than one category can be assigned to a protein.

Visualizations

Here, we present additional panels (supplementary to the main text) for visualizing 2D protein structure space using PaCMAP, for both normalized and unnormalized Geometricus representations, focusing on superCOG annotations.

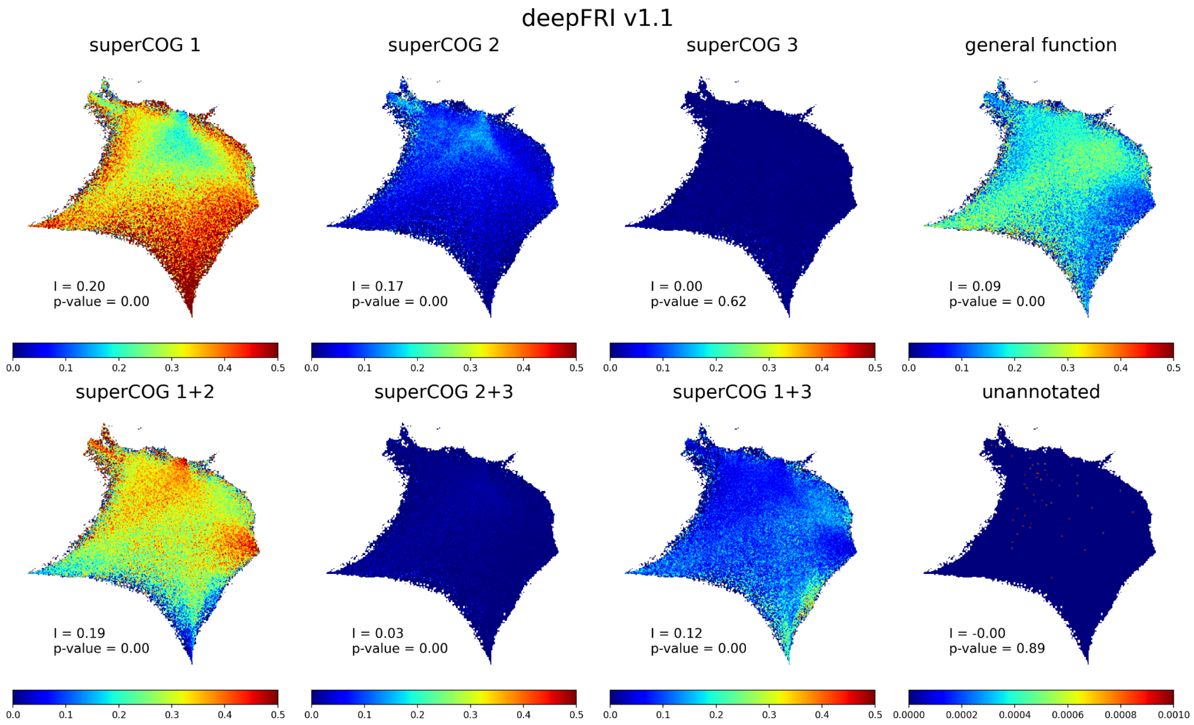

Supplementary Figure 41: The same as in Fig. 3b in the manuscript but for deepFRI v1.1.

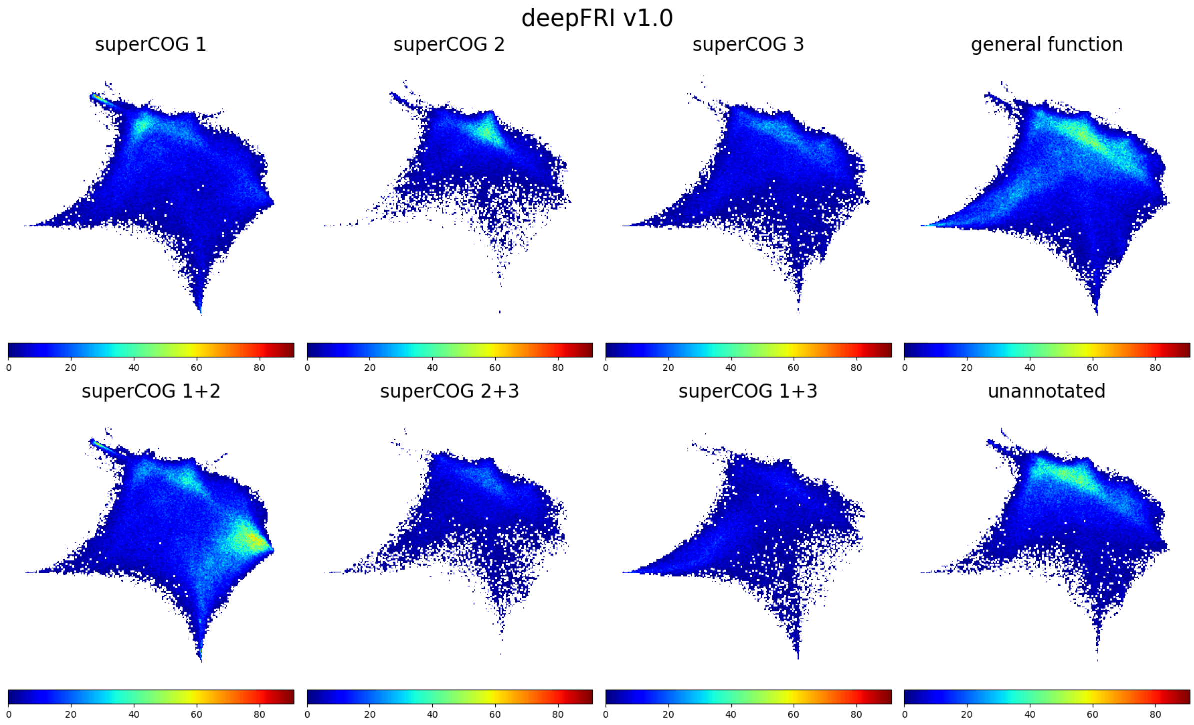

Supplementary Figure 42: The same as in Fig. 3b in the manuscript but the total number of structures (absolute values) is shown.

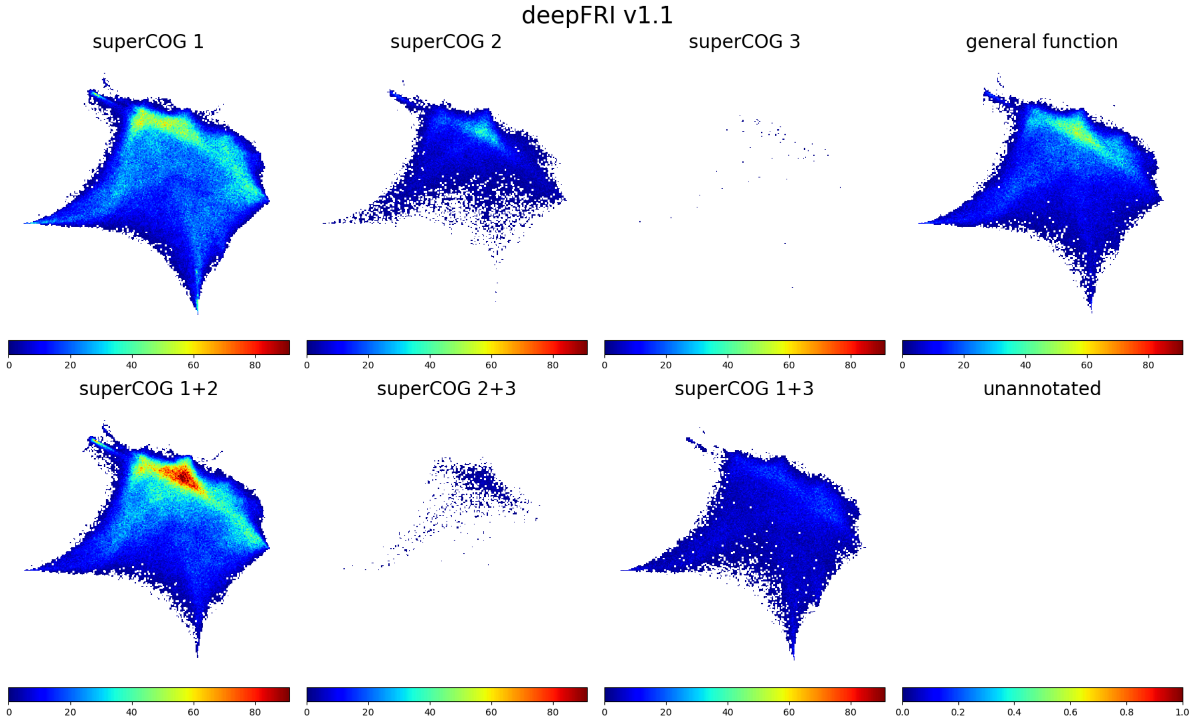

Supplementary Figure 43: The same as in Fig. 3b in the manuscript but the total number of structures (absolute values) is shown and deepFRI v1.1 is used.

Plots for unnormalized Geometricus representations

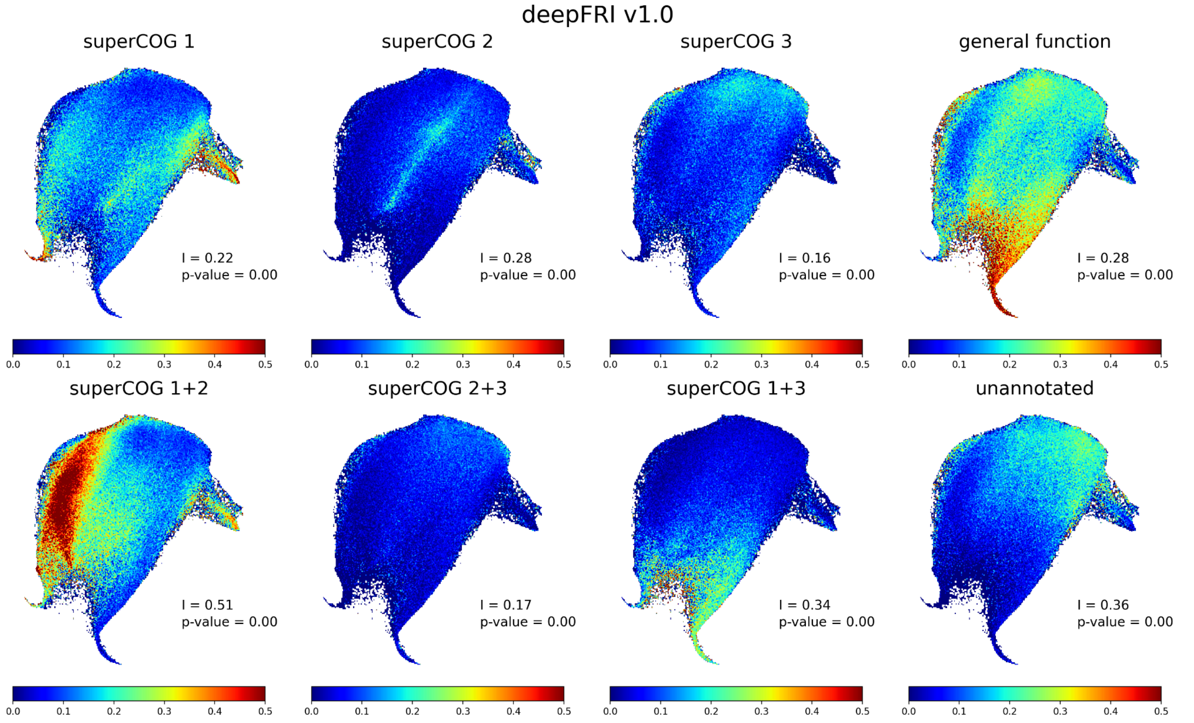

Supplementary Figure 44: The same as in Fig. 3b but for unnormalized Geometricus representations.

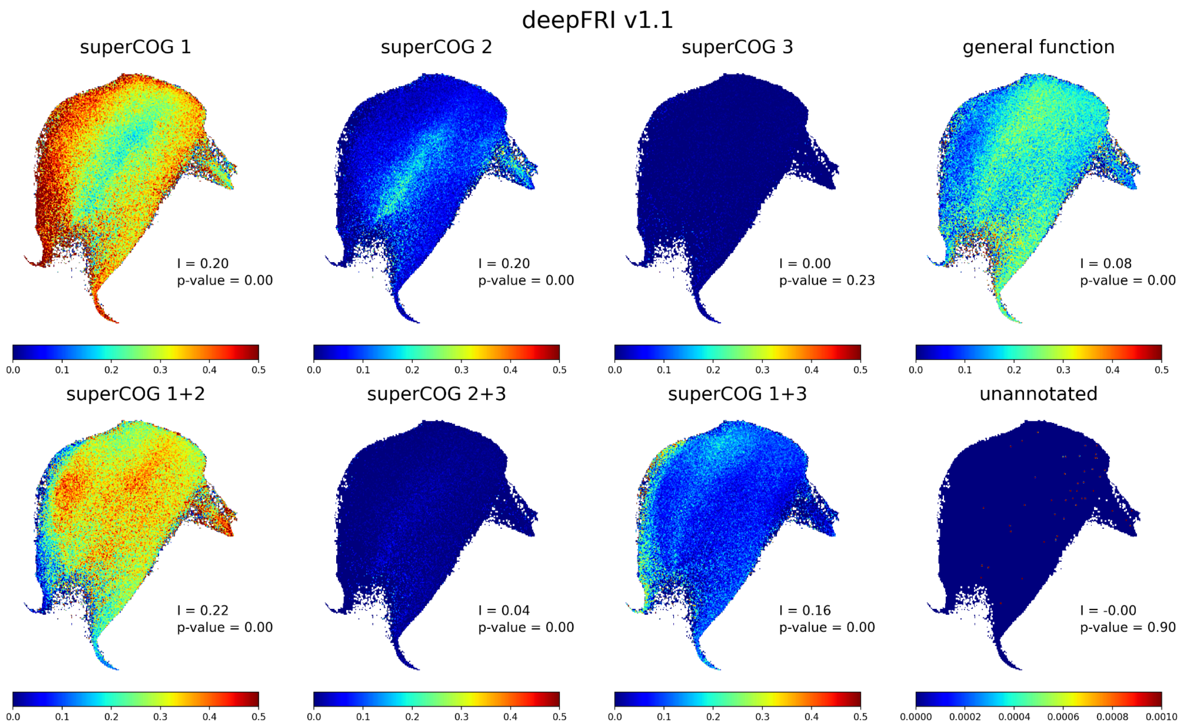

Supplementary Figure 45: The same as in Supplementary Figure 41 but for unnormalized Geometricus representations.

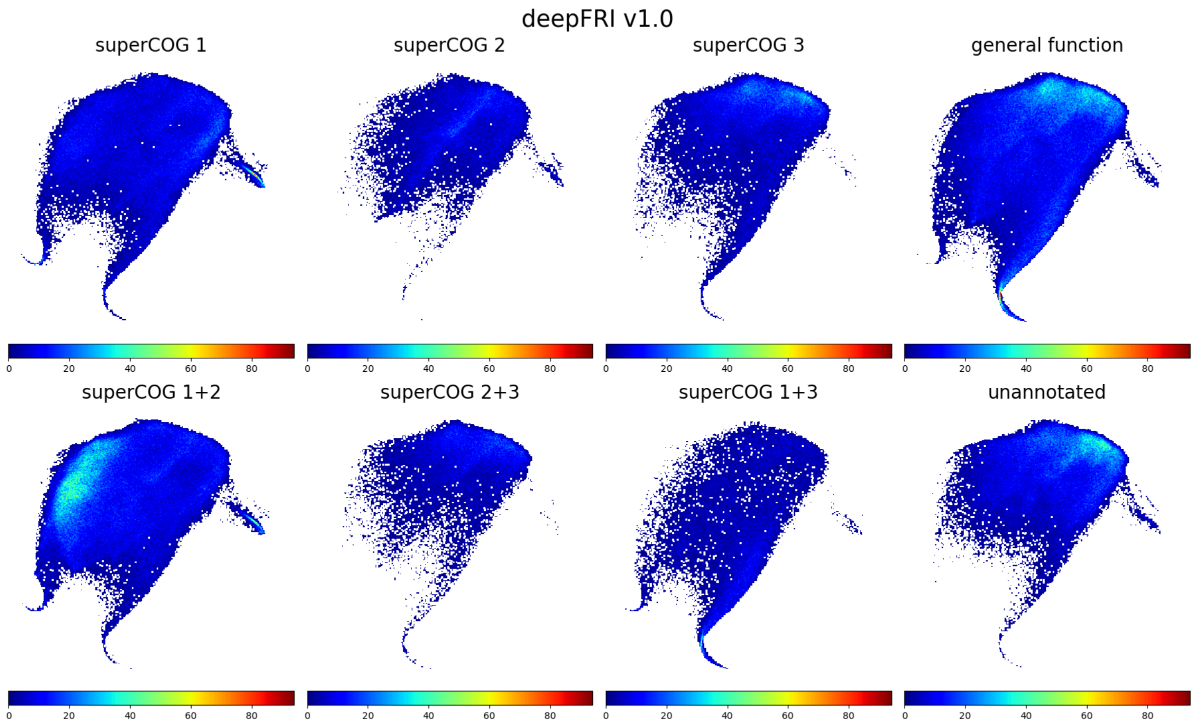

Supplementary Figure 46: The same as in Supplementary Figure 42 but for unnormalized Geometricus representations.

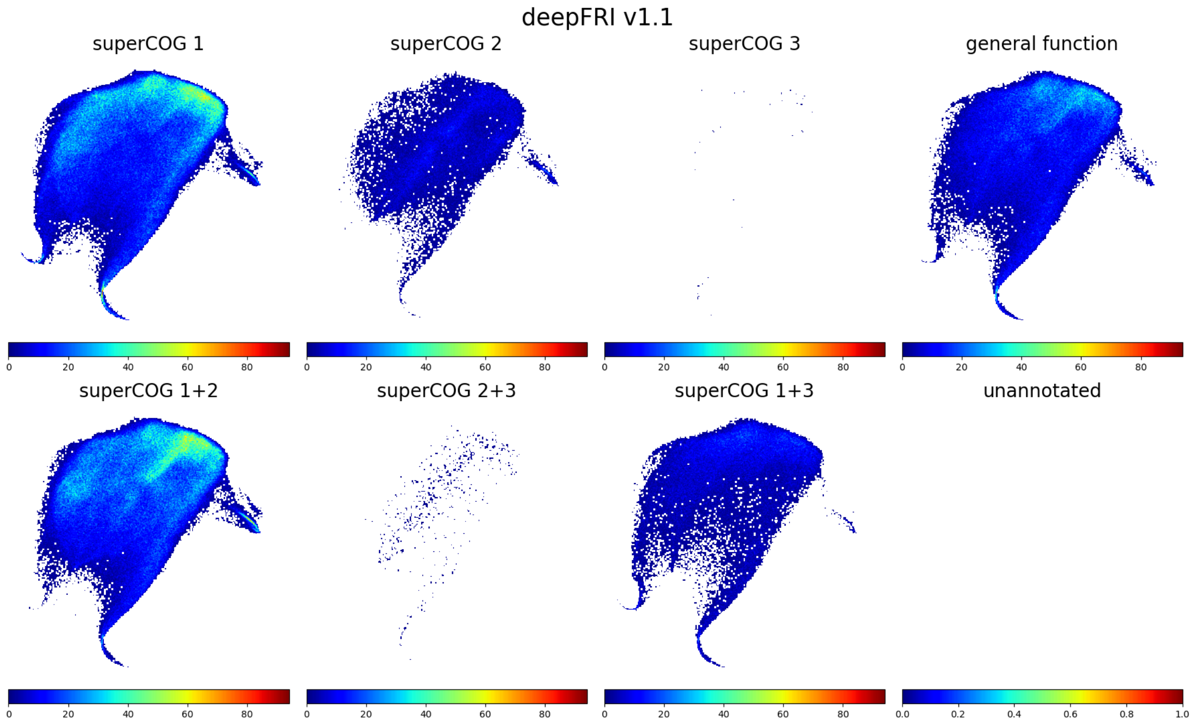

Supplementary Figure 47: The same as in Supplementary Figure 43 but for unnormalized Geometricus representations.

Top COG categories

Overview of functional annotation profiles for each database, represented as COG categories abundance, can be seen in Supplementary Figure 48. Except for MIP new folds and AFDB dark clusters, the annotation profile shows very similar proportions within a given deepFRI version.f

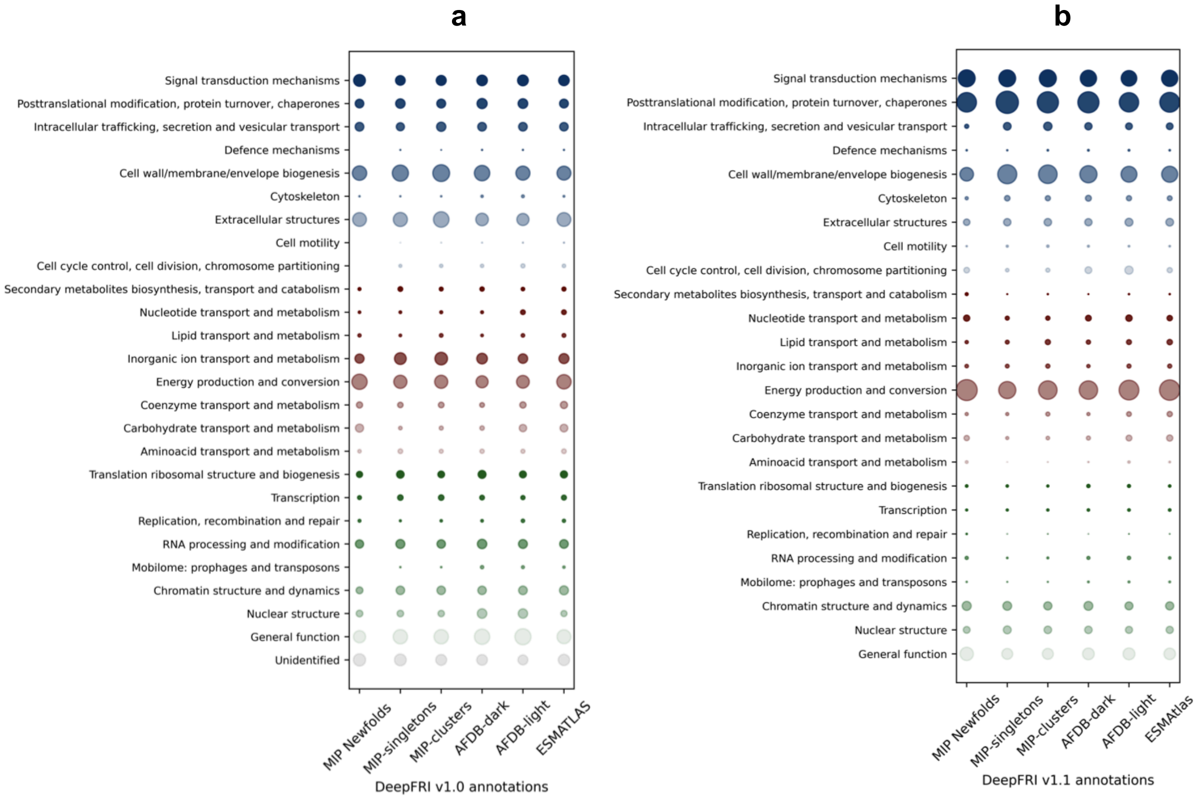

Supplementary Figure 48: Dot plot of proteins annotated by deepFRI v1.0 and v1.1 – **(a)** and **(b)** panels respectively. Annotations are represented as COG categories (with ≥ 1 COG per protein allowed) and dots represent relative abundance of COGs normalized to the protein count for each dataset.

Cluster heterogeneity

In Supplementary Figure 49-Supplementary Figure 55 other variations of Fig. 5b-c are shown. Only high-quality AFDB models have been taken into account. In Supplementary Figure 49-Supplementary Figure 51, restricting the analysis to structures within a specific size range or using the same number of structures per cluster range, which are two potential sources of bias, does not alter the overall trends. Agreement between Fig. 5b and Supplementary Figure 49 is high for AFDB + ESMAtlas and slightly worse for AFDB + ESMAtlas + MIP (especially for superCOG 1) but at the same time the statistics is poorer (much less structures per a given cluster size range). The locations in the structure space of proteins forming heterogeneous clusters are shown in Supplementary Figure 56-Supplementary Figure 57, which correlates with regions of high overlap between AFDB light, ESMAtlas, and MIP structures (see Fig. 1c).

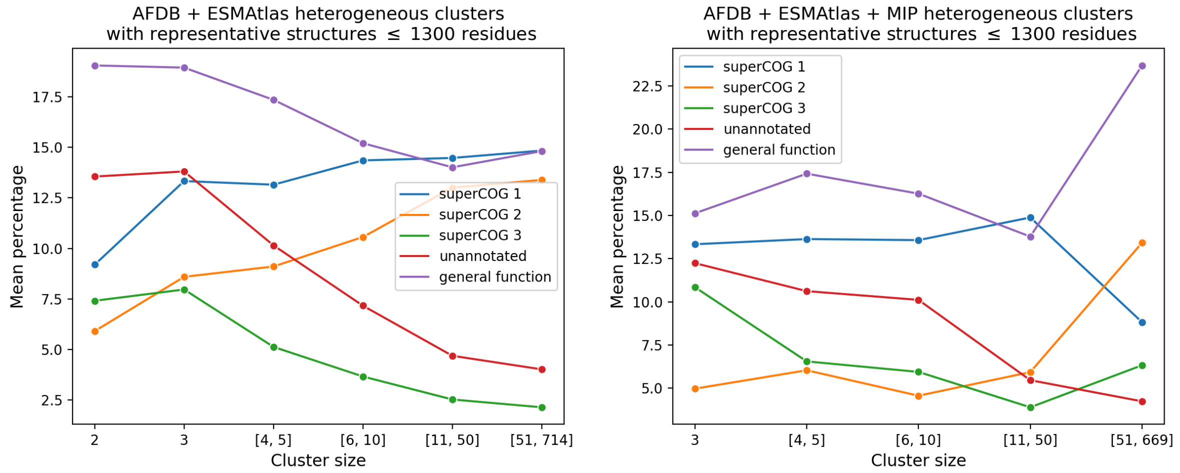

Supplementary Figure 49: Mean SuperCOG percentage for all structures in structurally heterogeneous clusters with high functional homogeneity (i.e., > 50% of structures annotated by one functional group). Percentage of clusters considered in the above plots: 35% and 28% for AFDB + ESMAtlas and AFDB + ESMAtlas + MIP respectively (compare with Fig. 5b in the manuscript where only representative structures are considered).

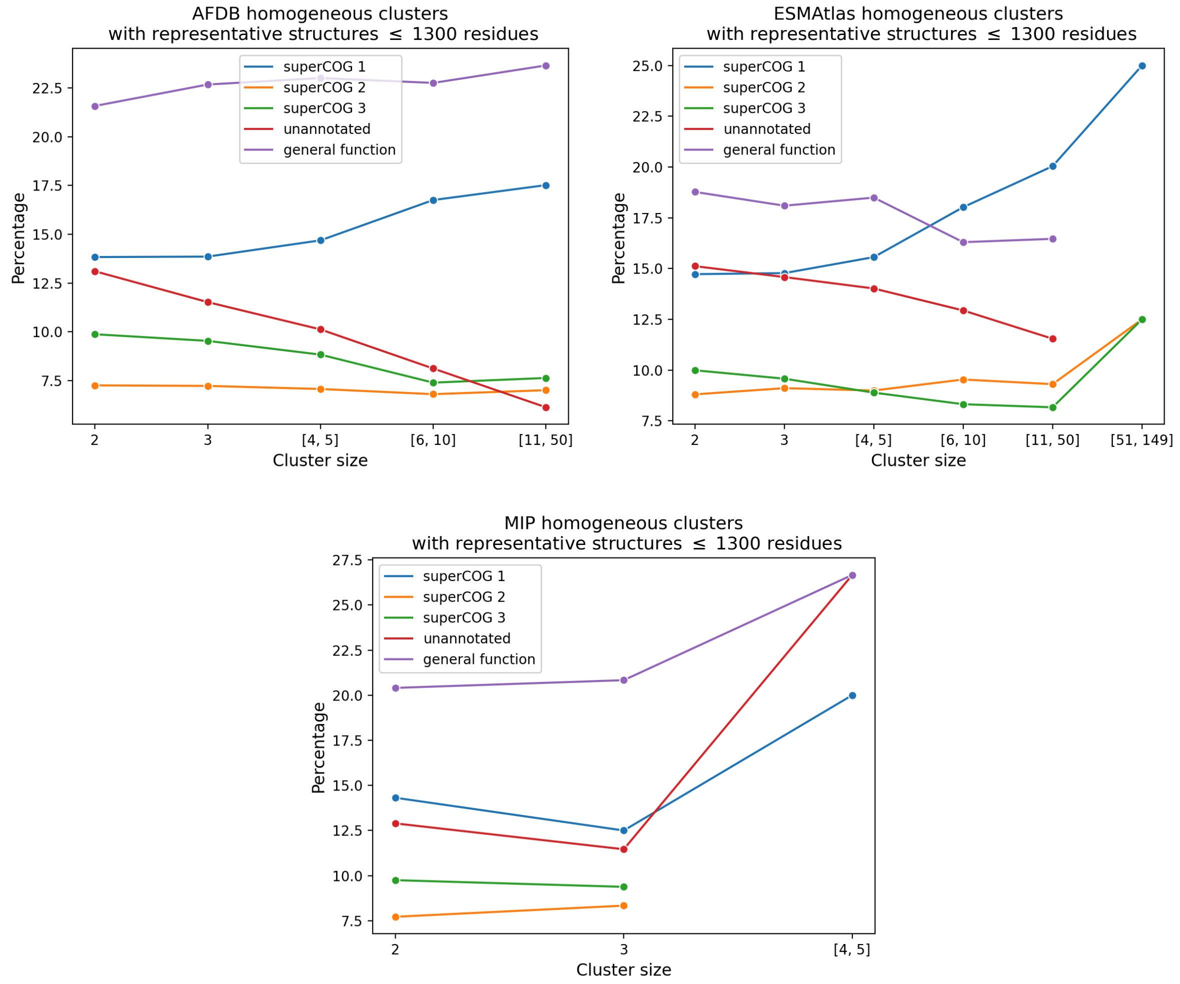

Supplementary Figure 50: The same as in Fig. 5b in the manuscript but for homogeneous clusters.

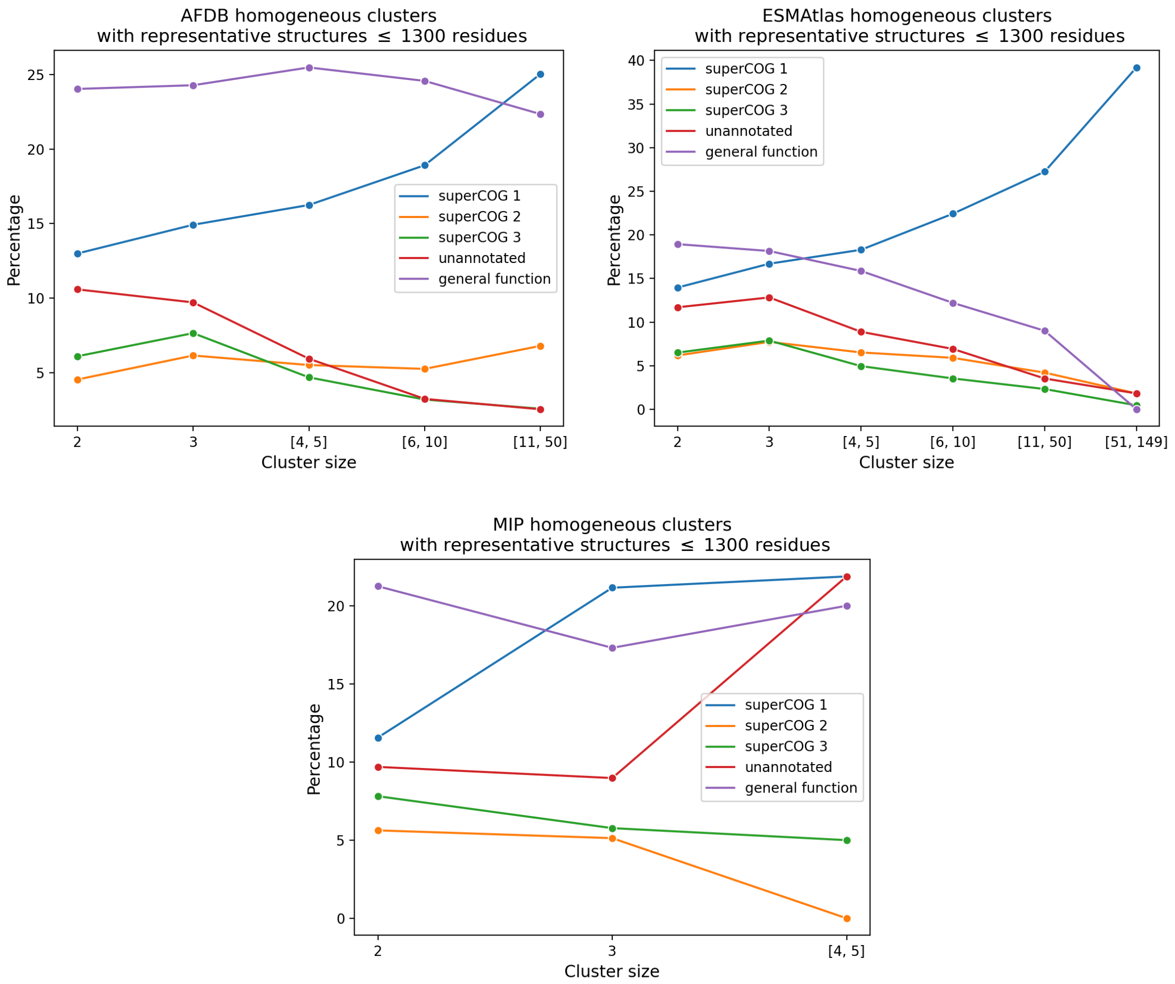

Supplementary Figure 51: The same as in Supplementary Figure 49 but for homogeneous clusters. Percentage of clusters considered in the above plots: 38%, 36%, 35% for AFDB, ESMAtlas and MIP respectively.

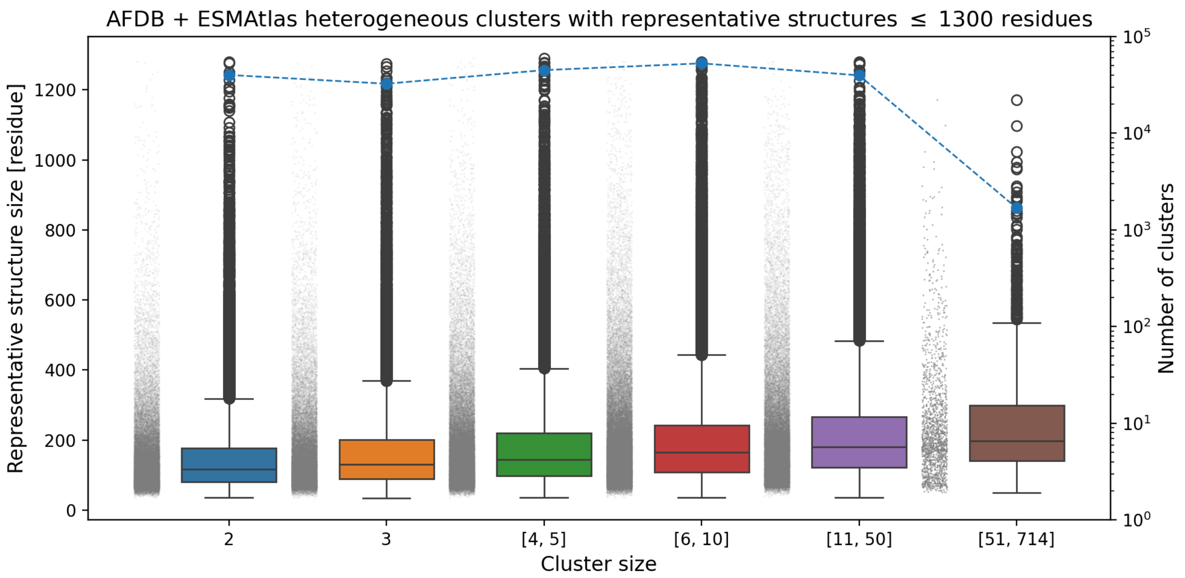

Supplementary Figure 52: The same as in Fig. 5c in the manuscript but for heterogeneous clusters having both AFDB and ESMAtlas structures. Data are presented as median values with interquartile

range (IQR); whiskers indicate variability outside the upper and lower quartiles, and circles represent outliers. Individual data points for each box are shown to the left.

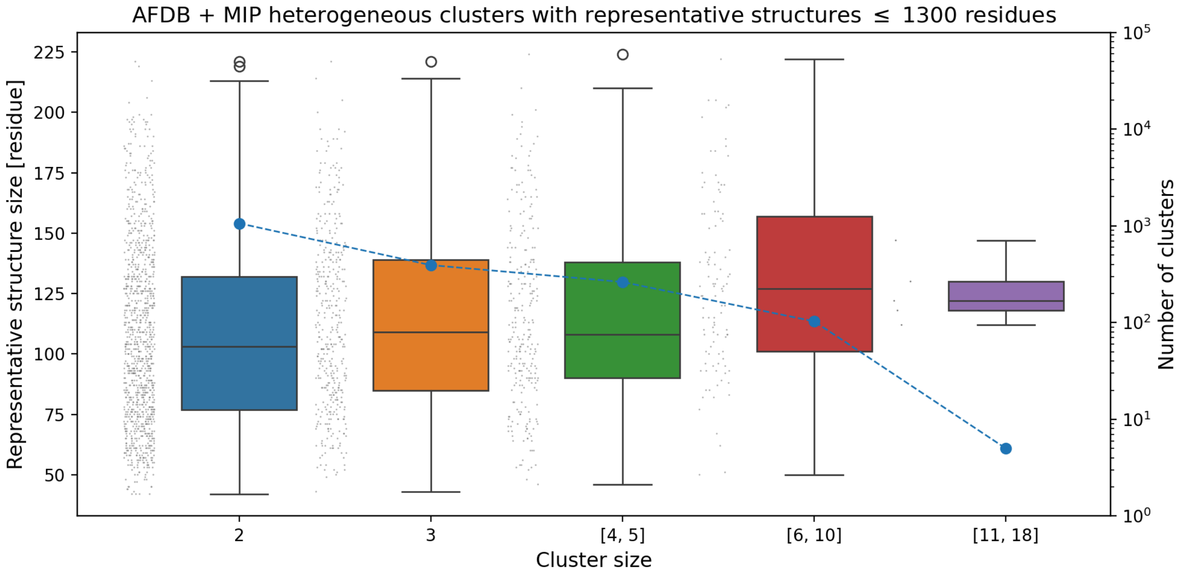

Supplementary Figure 53: The same as in Fig. 5c in the manuscript but for heterogeneous clusters having both AFDB and MIP structures. Data are presented as median values with interquartile range (IQR); whiskers indicate variability outside the upper and lower quartiles, and circles represent outliers. Individual data points for each box are shown to the left.

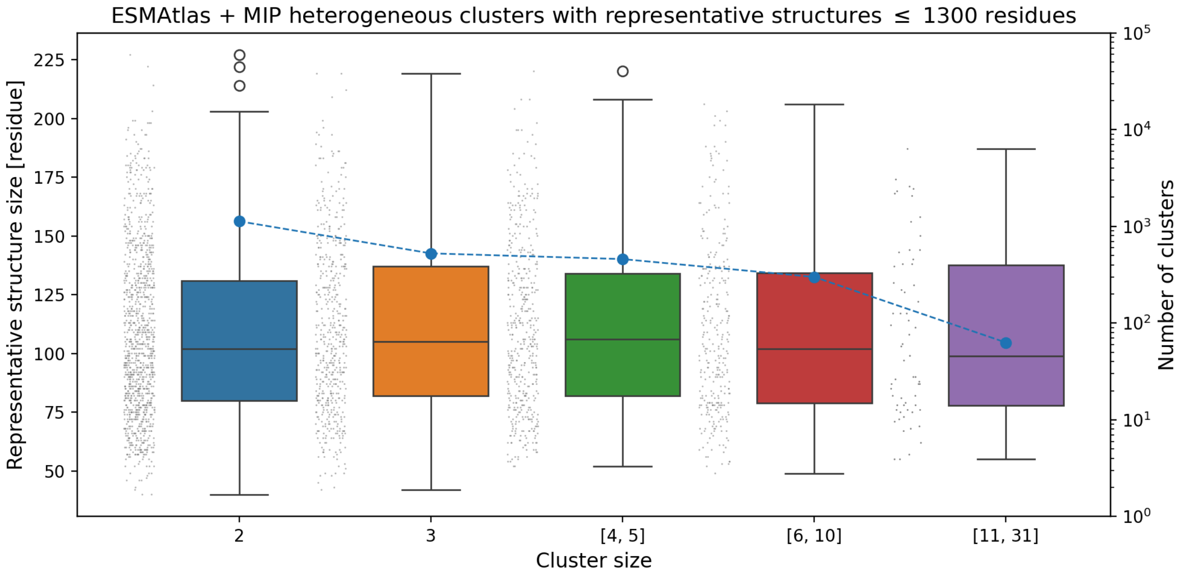

Supplementary Figure 54: The same as in Fig. 5c in the manuscript but for heterogeneous clusters having both ESMAtlas and MIP structures. Data are presented as median values with interquartile range (IQR); whiskers indicate variability outside the upper and lower quartiles, and circles represent outliers. Individual data points for each box are shown to the left.

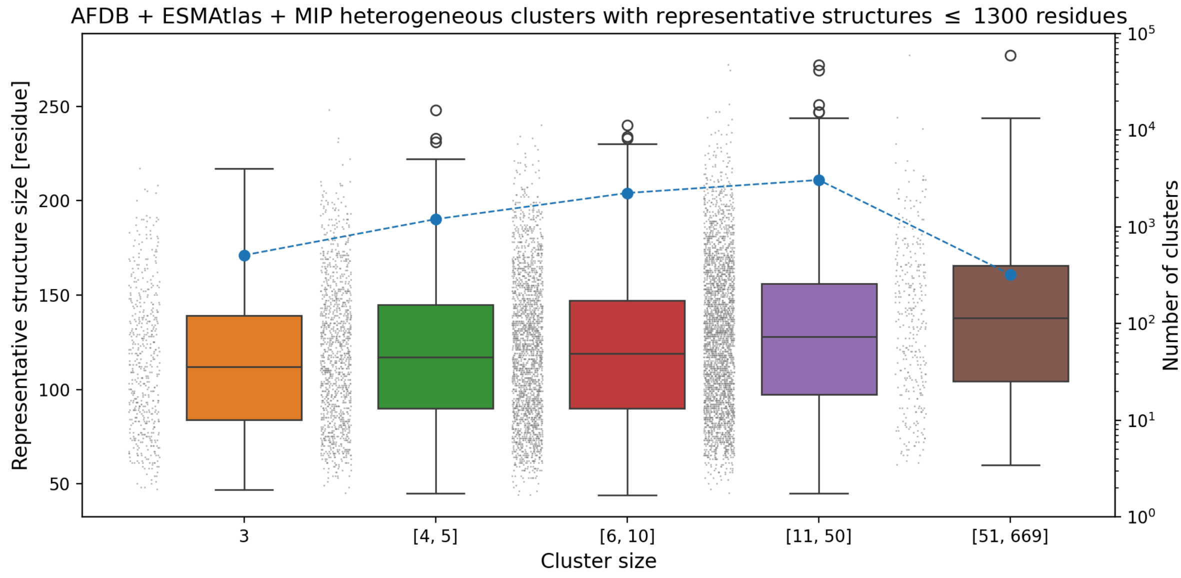

Supplementary Figure 55: The same as in Fig. 5c in the manuscript but for heterogeneous clusters having both AFDB, ESMAtlas, and MIP structures. Data are presented as median values with interquartile range (IQR); whiskers indicate variability outside the upper and lower quartiles, and circles represent outliers. Individual data points for each box are shown to the left.

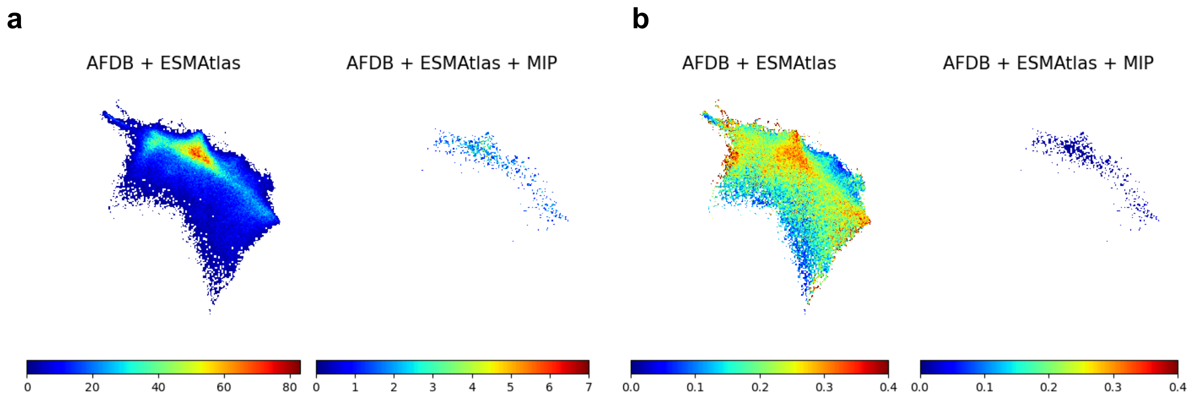

Supplementary Figure 56: (a): Number of representative structures originating from heterogeneous clusters of a given type (see titles). **(b):** Fraction of representative structures originating from heterogeneous clusters of a given type (see titles). Normalized Geometricus representations have been used. Only high-quality AF-DB models have been considered.

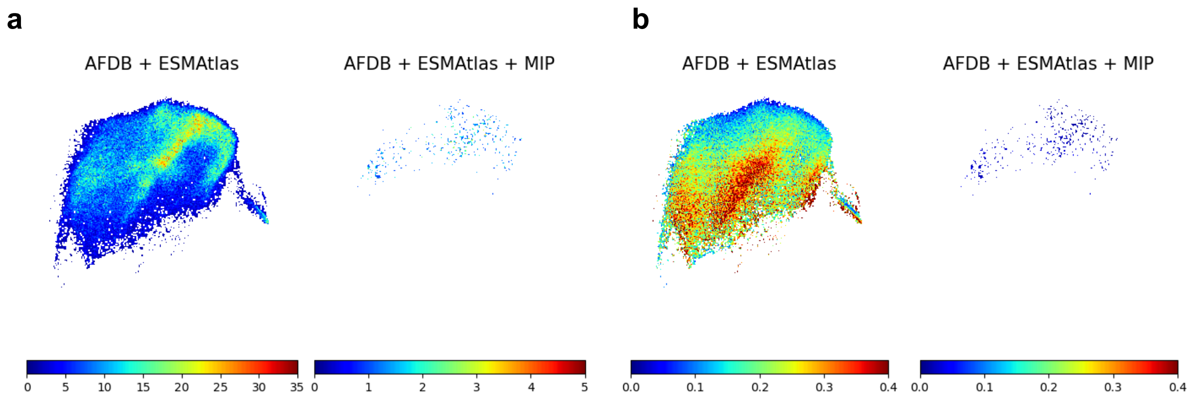

Supplementary Figure 57: The same as in Supplementary Figure 56 but for unnormalized Geometricus representations.

Supplementary Figure 58 highlights the largest representatives of homogeneous and heterogeneous clusters. Similarly to Fig. 2, we observe a high level of consistency across the structure space between different databases, although AFDB exhibits the greatest divergence among homogeneous groups. The heterogeneous clusters are located along the top edge of the space, with the AFDB + ESMAtlas group being more spread out and encompassing regions associated with ESMAtlas. Most of the structures in Supplementary Figure 58, represented in blue and magenta (groups A-F), resemble transmembrane or intramembrane proteins. This reflects both the ubiquity of these structures across databases and the diversity of cellular membranes among different organisms.

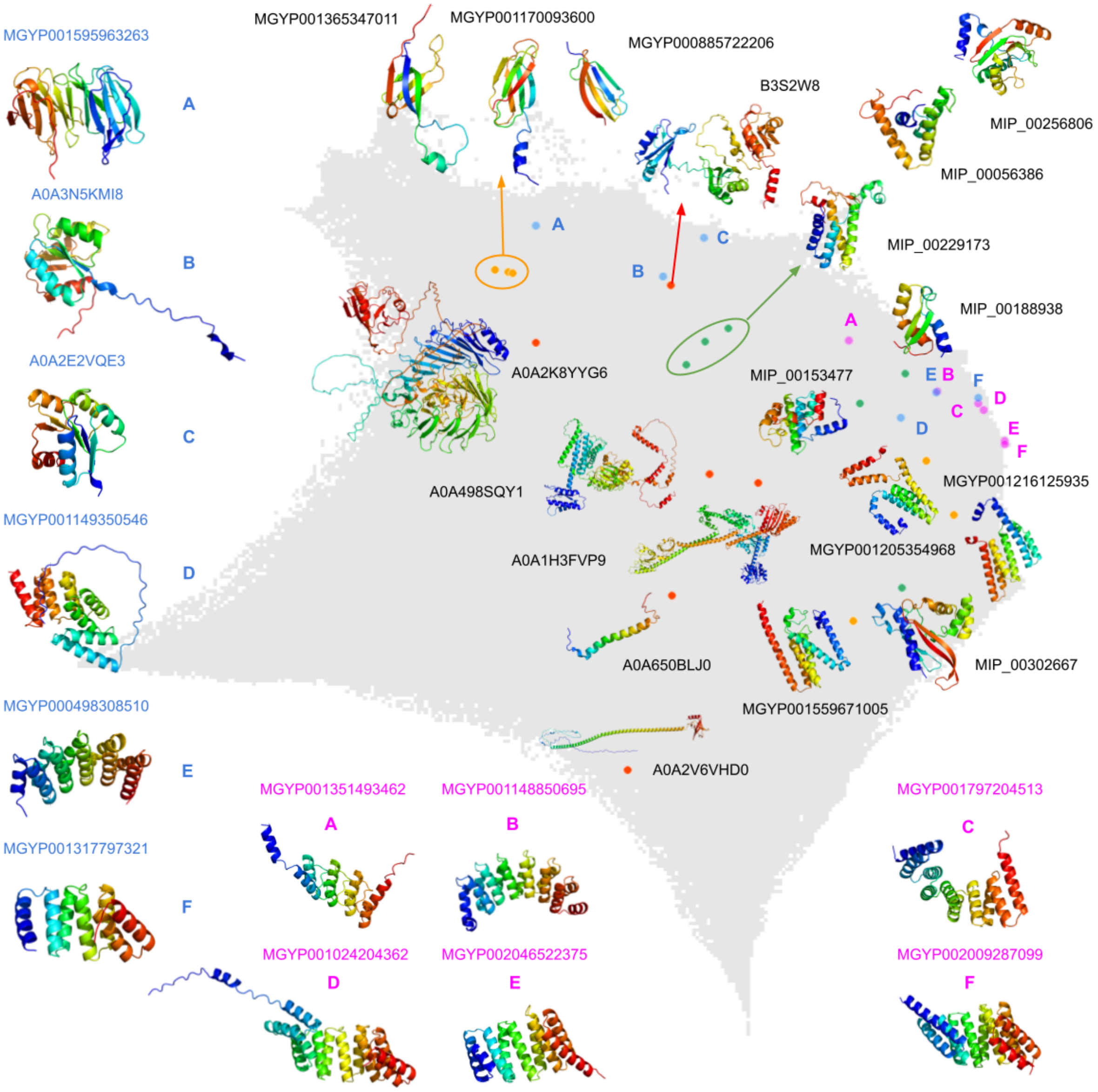

Supplementary Figure 58: Largest cluster representatives. Red, orange, and green dots represent AFDB, ESMAtlas, and MIP representatives of the top six largest homogeneous clusters,

with names and structure visualizations attached to the dots on the plot. Similarly, blue and magenta dots correspond to representatives of the top six largest heterogeneous clusters for AFDB + ESMAtlas and AFDB + ESMAtlas + MIP, respectively, with names and structure visualizations displayed outside the plot. For easier navigation, blue and magenta dots are labeled with letters.

Supplementary Figure 59 provides a quantitative comparison of functions within homogeneous and heterogeneous clusters. The SuperCOG 1+2 category is the most prevalent (>23%) in AFDB + ESMAtlas homogeneous clusters and is also the most common in ESMAtlas homogeneous clusters (>23%). In contrast, the AFDB homogeneous cluster is dominated by the general function group (>22%) and SuperCOG 1+2 (>20%). For AFDB and ESMAtlas singletons, general function is the most prevalent, followed by SuperCOG 1+2, whereas this order is reversed in MIP singletons.

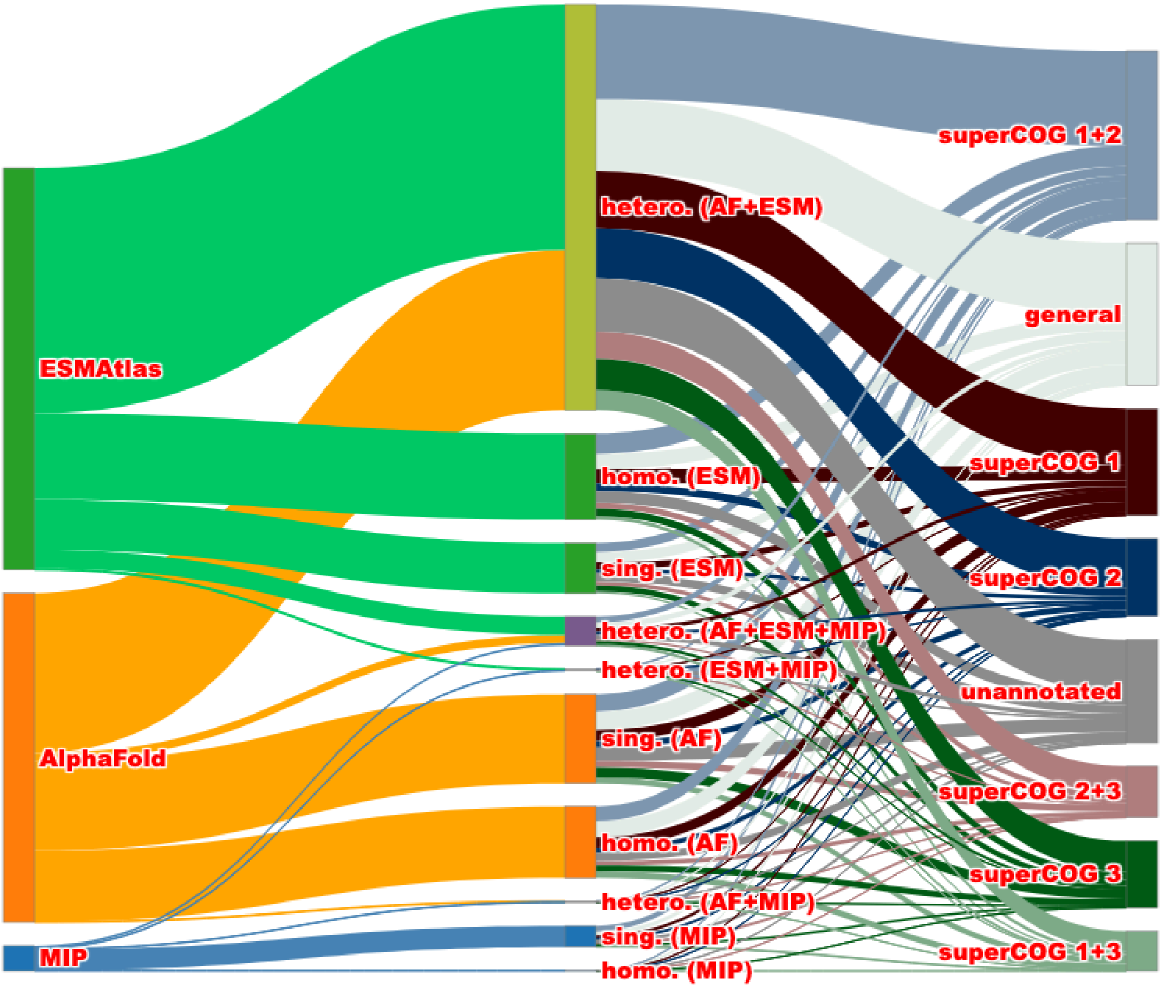

Supplementary Figure 59: Sankey diagram showing the number of AFDB, ESMAtlas, and MIP structures (first layer) forming different cluster types (second layer) and their functional annotation coverage (third layer).

Taxonomy analysis

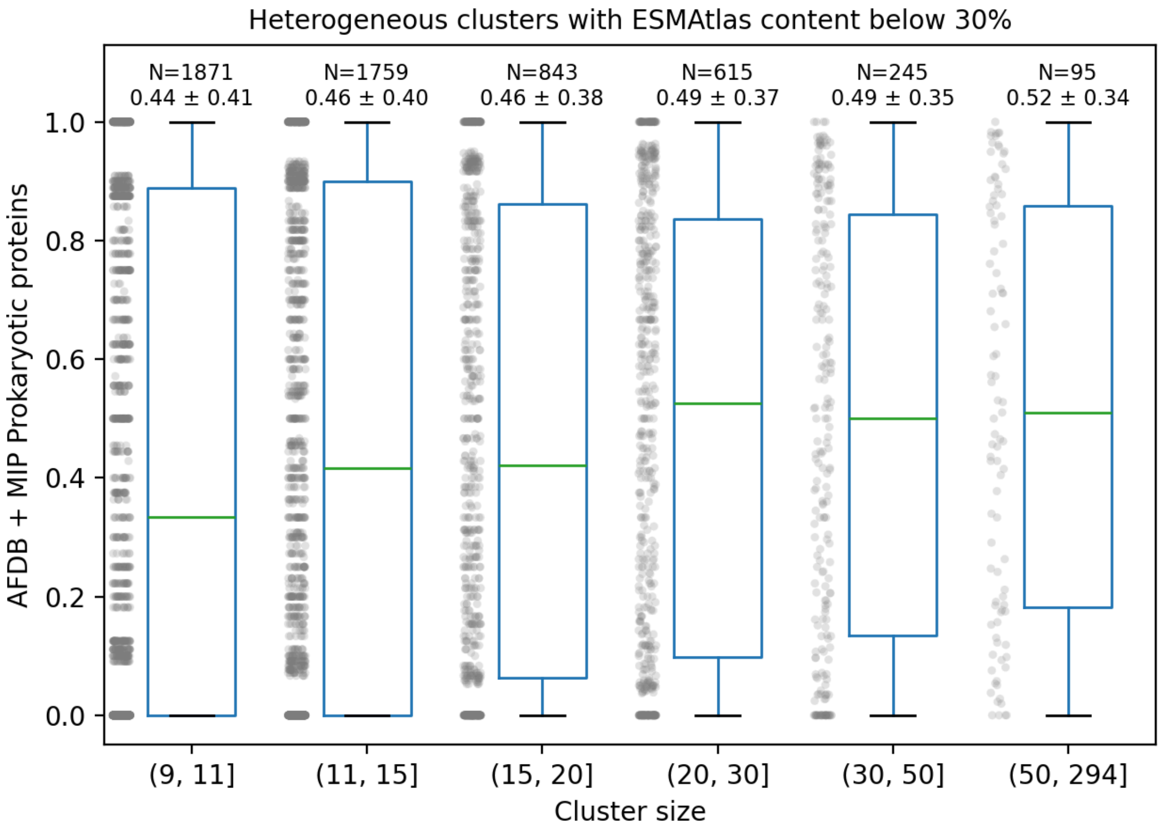

Supplementary Figure 60: Proportion of prokaryotic proteins with high quality AlphaFold models and MIP models in heterogeneous clusters with small ESMAtlas content (below 30%) as a function of cluster size. Data are presented as median values with interquartile range (IQR); whiskers indicate variability outside the upper and lower quartiles. Above each box is the number of data points and mean +/- standard deviation. Individual data points for each box are shown to the left.

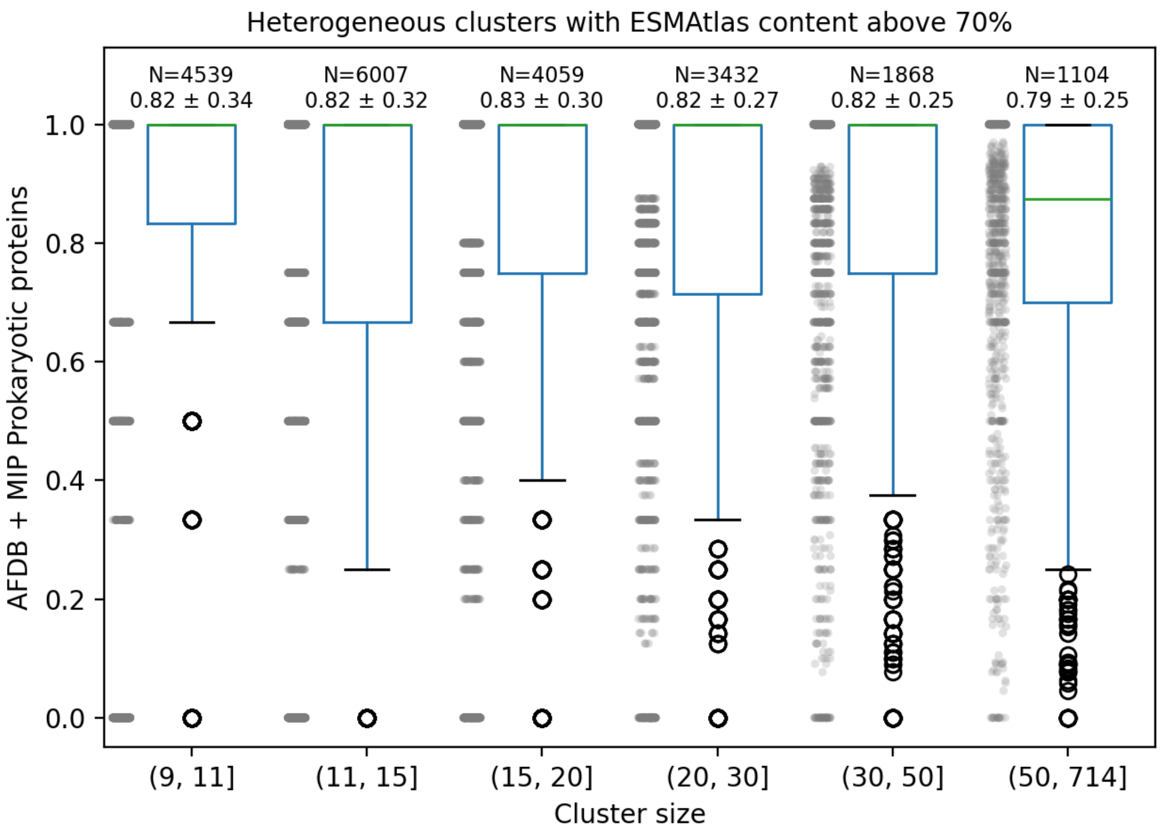

Supplementary Figure 61: Proportion of prokaryotic proteins with high quality AlphaFold models and MIP models in heterogeneous clusters with large ESMAtlas content (above 70%) as a function of cluster size. Data are presented as median values with interquartile range (IQR); whiskers indicate variability outside the upper and lower quartiles, and circles represent outliers. Above each box is the number of data points and mean +/- standard deviation. Individual data points for each box are shown to the left.

Supplementary Table 6: Number of proteins from each database for cluster representatives depicted in Fig. 6c.

	MGYP003387535279	MGYP001416922367	U6GJL0	A0A6A5UDC9
AFDB light	55	71	97	99
AFDB dark	0	1	0	0
ESMAtlas	78	71	48	348
MIP	0	0	0	0

Supplementary Table 7: Taxonomic composition of cluster representatives used for entropy calculation (Fig. 6c).

	MGYP003387535279	MGYP001416922367	U6GJL0	A0A6A5UDC9
Plants and Fungi	20	30	34	48
Bacteria	5	12	20	24

Invertebrates	16	21	31	17
Vertebrates	9	4	7	5
Primates	0	2	2	2
Environmental samples	1	0	0	1
Rodents	1	0	1	1
Mammals	3	1	2	1
Unknown	0	2	0	0

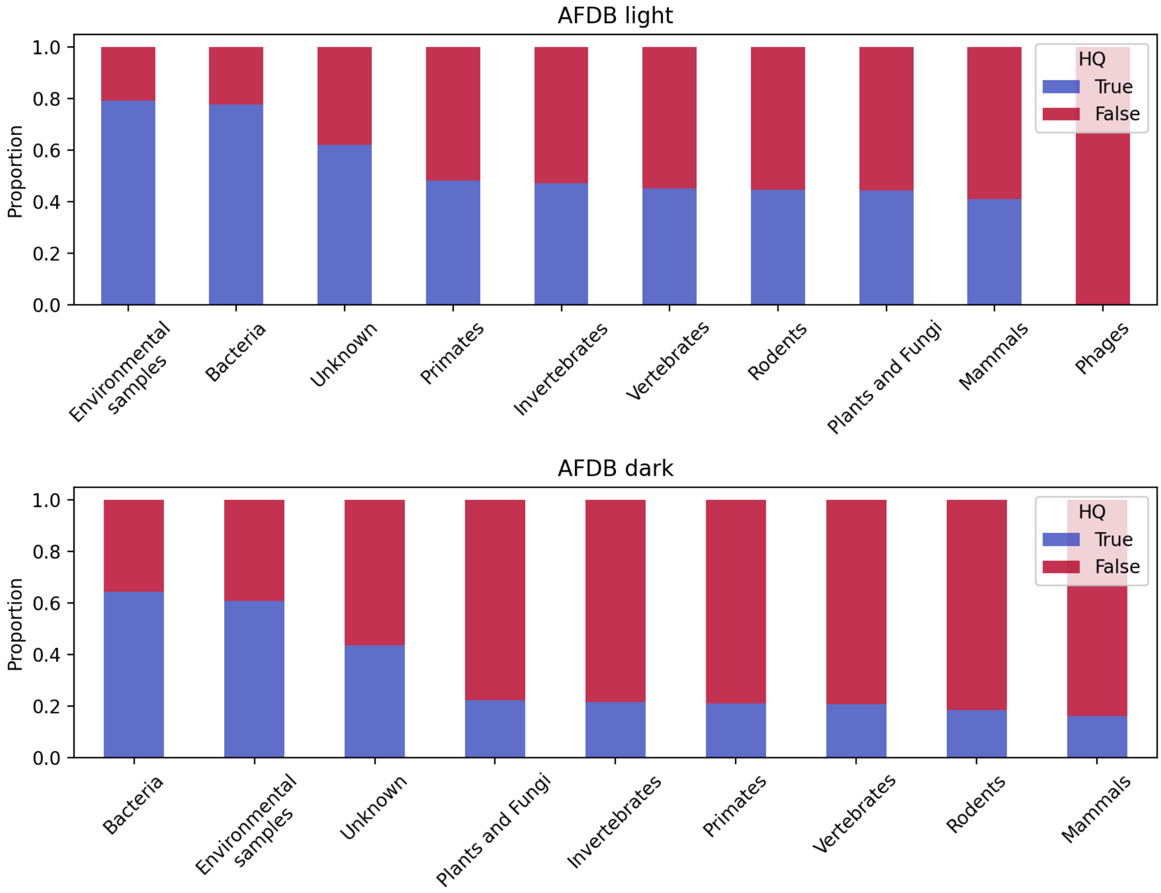

Supplementary Figure 62: Proportion of high quality AlphaFold predictions (HQ=True i.e. pLDDT > 70) stratified by taxonomic group for light and dark AFDB cluster representatives.

References

1. Barrio-Hernandez,I., Yeo,J., Jänes,J., Mirdita,M., Gilchrist,C.L.M., Wein,T., Varadi,M., Velankar,S., Beltrao,P. and Steinegger,M. (2023) Clustering predicted structures at the scale of the known protein universe. *Nature*, **622**, 637–645.

2. Koehler Leman,J., Szczerbiak,P., Renfrew,P.D., Gligorijevic,V., Berenberg,D., Vatanen,T., Taylor,B.C., Chandler,C., Janssen,S., Pataki,A., *et al.* (2023)

Sequence-structure-function relationships in the microbial protein universe. *Nat. Commun.*, **14**, 1–11.

3. Suzek,B.E., Wang,Y., Huang,H., McGarvey,P.B., Wu,C.H. and UniProt Consortium (2015) UniRef clusters: a comprehensive and scalable alternative for improving sequence similarity searches. *Bioinformatics*, **31**, 926–932.

4. Gligorijević,V., Renfrew,P.D., Kosciolek,T., Leman,J.K., Berenberg,D., Vatanen,T., Chandler,C., Taylor,B.C., Fisk,I.M., Vlamakis,H., *et al.* (2021) Structure-based protein function prediction using graph convolutional networks. *Nat. Commun.*, **12**, 1–14.
