# Supplementary material for: Large protein databases reveal structural complementarity and functional locality
Source: Nat Commun. 2025 Aug 25;16:7925. doi: 10.1038/s41467-025-63250-3 (PMC12378989; doi:10.1038/s41467-025-63250-3)
Supplement: Supplementary file 2 — Reporting Summary [file 41467_2025_63250_MOESM2_ESM.pdf]

nature portfolio

Corresponding author(s): Tomasz Kosciolek

Last updated by author(s): Jul 18, 2025

Reporting Summary

Nature Portfolio wishes to improve the reproducibility of the work that we publish. This form provides structure for consistency and transparency in reporting. For further information on Nature Portfolio policies, see our Editorial Policies and the Editorial Policy Checklist.

Statistics

For all statistical analyses, confirm that the following items are present in the figure legend, table legend, main text, or Methods section.

n/a	Confirmed	
☐	☒	The exact sample size (*n*) for each experimental group/condition, given as a discrete number and unit of measurement
☒	☐	A statement on whether measurements were taken from distinct samples or whether the same sample was measured repeatedly
☐	☒	The statistical test(s) used AND whether they are one- or two-sided<br>*Only common tests should be described solely by name; describe more complex techniques in the Methods section.*
☐	☒	A description of all covariates tested
☐	☒	A description of any assumptions or corrections, such as tests of normality and adjustment for multiple comparisons
☐	☒	A full description of the statistical parameters including central tendency (e.g. means) or other basic estimates (e.g. regression coefficient) AND variation (e.g. standard deviation) or associated estimates of uncertainty (e.g. confidence intervals)
☐	☒	For null hypothesis testing, the test statistic (e.g. *F*, *t*, *r*) with confidence intervals, effect sizes, degrees of freedom and *P* value noted<br>*Give P values as exact values whenever suitable.*
☒	☐	For Bayesian analysis, information on the choice of priors and Markov chain Monte Carlo settings
☒	☐	For hierarchical and complex designs, identification of the appropriate level for tests and full reporting of outcomes
☒	☐	Estimates of effect sizes (e.g. Cohen's *d*, Pearson's *r*), indicating how they were calculated

Our web collection on statistics for biologists contains articles on many of the points above.

Software and code

Policy information about availability of computer code

Data collection	AFDB clusters and hclust30: https://foldcomp.steineggerlab.workers.dev/<br>MIP: https://zenodo.org/records/6611431<br>Figshare repository https://dx.doi.org/10.6084/m9.figshare.27203073<br>The data are also available in our GitHub repository: https://github.com/Tomasz-Lab/protein-structure-landscape (release v1.0)
Data analysis	The code required to reproduce our analysis is available in our Figshare repository https://dx.doi.org/10.6084/m9.figshare.27203073 and GitHub repository: https://github.com/Tomasz-Lab/protein-structure-landscape (release v1.0). Only open-source third party software was used and are described in Methods section.

For manuscripts utilizing custom algorithms or software that are central to the research but not yet described in published literature, software must be made available to editors and reviewers. We strongly encourage code deposition in a community repository (e.g. GitHub). See the Nature Portfolio guidelines for submitting code & software for further information.

Data

Policy information about availability of data

All manuscripts must include a data availability statement. This statement should provide the following information, where applicable:

- Accession codes, unique identifiers, or web links for publicly available datasets
- A description of any restrictions on data availability
- For clinical datasets or third party data, please ensure that the statement adheres to our policy

Source data required to reproduce all figures in the article and Supplementary Information, lists of clusters and singletons obtained from Foldseek, relevant metadata (such as structure space coordinates, functional annotations, and the number of structures from each dataset), as well as Figure 6b and Supplementary Figure 59 in HTML format can be found in our Figshare repository https://dx.doi.org/10.6084/m9.figshare.27203073. The data mentioned above (except source data) are also available in our GitHub repository: https://github.com/Tomasz-Lab/protein-structure-landscape (release v1.0). Unless otherwise stated, all data supporting the results of this study can be found in the article, supplementary, and source data files.

Research involving human participants, their data, or biological material

Policy information about studies with human participants or human data. See also policy information about sex, gender (identity/presentation), and sexual orientation and race, ethnicity and racism.

Reporting on sex and gender	N/A
Reporting on race, ethnicity, or other socially relevant groupings	N/A
Population characteristics	N/A
Recruitment	N/A
Ethics oversight	N/A

Note that full information on the approval of the study protocol must also be provided in the manuscript.

Field-specific reporting

Please select the one below that is the best fit for your research. If you are not sure, read the appropriate sections before making your selection.

- [x] Life sciences
- [ ] Behavioural & social sciences
- [ ] Ecological, evolutionary & environmental sciences

For a reference copy of the document with all sections, see nature.com/documents/nr-reporting-summary-flat.pdf

Life sciences study design

All studies must disclose on these points even when the disclosure is negative.

Sample size	Information on sample sizes is provided in the manuscript text and included in each Figure of the manuscript and in the Supplement
Data exclusions	Information on data exclusions is provided in the Methods section of the manuscript
Replication	Replication of individual 3D structure predictions is extensively discussed in the scientific literature and was beyond the scope of this work.
Randomization	used where appropriate; Information on randomization is provided in the Methods section of the manuscript
Blinding	used where appropriate at the data analysis stage; specific blinding cases are provided in the Methods section of the manuscript

Reporting for specific materials, systems and methods

We require information from authors about some types of materials, experimental systems and methods used in many studies. Here, indicate whether each material, system or method listed is relevant to your study. If you are not sure if a list item applies to your research, read the appropriate section before selecting a response.

Materials & experimental systems

n/a	Involved in the study
☒	☐ Antibodies
☒	☐ Eukaryotic cell lines
☒	☐ Palaeontology and archaeology
☒	☐ Animals and other organisms
☒	☐ Clinical data
☒	☐ Dual use research of concern
☒	☐ Plants

Methods

n/a	Involved in the study
☒	☐ ChIP-seq
☒	☐ Flow cytometry
☒	☐ MRI-based neuroimaging

Plants

Seed stocks	N/A
Novel plant genotypes	N/A
Authentication	N/A
